# Aim32 is a dual-localized 2Fe-2S mitochondrial protein that functions in redox quality control

**DOI:** 10.1016/j.jbc.2021.101135

**Published:** 2021-08-28

**Authors:** Danyun Zhang, Owen R. Dailey, Daniel J. Simon, Kamilah Roca-Datzer, Yasaman Jami-Alahmadi, Mikayla S. Hennen, James A. Wohlschlegel, Carla M. Koehler, Deepa V. Dabir

**Affiliations:** 1 Department of Chemistry and Biochemistry, UCLA, Los Angeles, California, USA; 2 Department of Biology, Loyola Marymount University, Los Angeles, California, USA; 3 Department of Biological Chemistry, UCLA, Los Angeles, California, USA; 4 Jonsson Comprehensive Cancer Center, UCLA, Los Angeles, California, USA

**Keywords:** mitochondria, mitochondrial transport, redox regulation, thioredoxin, thiol, disulfide, protein import, 2D-DGE, two-dimensional diagonal gel electrophoresis, Aim32, altered inheritance of mitochondria 32, BN-PAGE, Blue native PAGE, CCCP, carbonyl cyanide m-chlorophenyl hydrazone, CNAP, consecutive nondenaturing affinity purification, Cyt *c*, cytochrome *c*, DiSC_3_(5), 3,3′-dipropylthiadicarbocyanine iodide, ER, endoplasmic reticulum, EtBr, ethidium bromide, HU, hydroxyurea, IAA, iodoacetamide, IM, inner membrane, IMS, intermembrane space, MIA, mitochondrial intermembrane space assembly, mmPEG, methyl-PEG-24-maleimide, MPP, matrix processing peptidase, PQ, primaquine, ROS, reactive oxygen species, TEV, tobacco etch virus, TLF, thioredoxin-like ferredoxin, Vec, empty vector, ΔΨ, membrane potential

## Abstract

Yeast is a facultative anaerobe and uses diverse electron acceptors to maintain redox-regulated import of cysteine-rich precursors via the mitochondrial intermembrane space assembly (MIA) pathway. With the growing diversity of substrates utilizing the MIA pathway, understanding the capacity of the intermembrane space (IMS) to handle different types of stress is crucial. We used MS to identify additional proteins that interacted with the sulfhydryl oxidase Erv1 of the MIA pathway. Altered inheritance of mitochondria 32 (Aim32), a thioredoxin-like [2Fe-2S] ferredoxin protein, was identified as an Erv1-binding protein. Detailed localization studies showed that Aim32 resided in both the mitochondrial matrix and IMS. Aim32 interacted with additional proteins including redox protein Osm1 and protein import components Tim17, Tim23, and Tim22. Deletion of Aim32 or mutation of conserved cysteine residues that coordinate the Fe-S center in Aim32 resulted in an increased accumulation of proteins with aberrant disulfide linkages. In addition, the steady-state level of assembled TIM22, TIM23, and Oxa1 protein import complexes was decreased. Aim32 also bound to several mitochondrial proteins under nonreducing conditions, suggesting a function in maintaining the redox status of proteins by potentially targeting cysteine residues that may be sensitive to oxidation. Finally, Aim32 was essential for growth in conditions of stress such as elevated temperature and hydroxyurea, and under anaerobic conditions. These studies suggest that the Fe-S protein Aim32 has a potential role in general redox homeostasis in the matrix and IMS. Thus, Aim32 may be poised as a sensor or regulator in quality control for a broad range of mitochondrial proteins.

Mitochondrial biogenesis depends on coordinated translocation machineries to correctly target and fold imported cytosolic proteins. In the intermembrane space (IMS), a subset of proteins acquires disulfide bonds in an oxidative folding pathway (1, 2, 3, 4). The mitochondrial intermembrane space assembly (MIA) pathway consists of the oxidoreductase Mia40s and sulfhydryl oxidase Erv1 that coordinate the ixnsertion of disulfide bonds into substrate proteins (3, 4, 5, 6, 7). Substrates typically have a CX3C (i.e., the small Tim proteins) or CX9C motif (i.e., proteins such as Cox19 that may have a role in complex IV assembly and cross the membrane in a reduced and unfolded state). Mia40 serves as an import receptor and mediates disulfide bond insertion (8, 9). Erv1 then reoxidizes Mia40 (9), and the electrons are passed to a variety of terminal electron acceptors including O_2_ and cytochrome (cyt) *c* (10, 11). In yeast, the MIA pathway functions under anaerobic conditions with the fumarate/Osm1 couple (12). However, *Saccharomyces cerevisiae* seems to have additional terminal electron acceptors because strains deleted for *OSM1* and *CYC1* were still viable under anaerobic conditions (12).

In contrast to the endoplasmic reticulum (ER), the MIA pathway lacks a bona fide disulfide isomerase. With model substrate Cox19, Mia40 is necessary and sufficient to introduce both disulfide bonds in a coordinated process (13). However, Mia40 is a peculiar oxidoreductase in that it has no isomerase activity (14). Within the IMS, glutathione may have proofreading capacity, preventing the formation of nonproductive intermediates (15); however, these studies were done *in vitro* and may not reflect the scenario *in vivo* (9, 16). Because yeast is a facultative anaerobe, it may use different electron acceptors, such as thioredoxins, glutaredoxins, and iron–sulfur (Fe/S) cluster proteins, to sustain oxidative folding across diverse environmental conditions.

Redox control within the mitochondrion is essential for the proper functioning of the organelle. The thiol-disulfide balance in the matrix and IMS is quite different; the mitochondrial matrix is considerably more reducing than the mitochondrial IMS (17). Redox homeostasis in the matrix and IMS is maintained independently (18, 19). In the matrix, glutathione reductase and mitochondrial thioredoxin reductase are key regulators (20). Sod2 is a matrix antioxidant that prevents oxidative damage to proteins by neutralizing reactive oxygen species (ROS) (21). Any changes in the redox state in one compartment may affect the other as has been shown for the ER where inhibiting protein folding has a major effect on overall cellular redox due to the operation of the refolding pathway (22).

Altered inheritance of mitochondria 32 (Aim32) was first reported in a study that assessed the mode of action of the antimalarial drug (primaquine [PQ]) and demonstrated to counteract PQ-induced oxidative stress in the absence of *SOD2* (23). More recently, it was shown by Stegmaier *et al*. that Aim32 is a soluble mitochondrial matrix protein, and a prototype of the widely distributed class of Fe/S proteins bearing a thioredoxin-like ferredoxin (TLF) domain with a bis-histidinyl coordinated [2Fe-2S] cluster (24). They further postulated a general role for Aim32 in the degradation of allelochemicals, such as flavonoids and phenolic compounds. However, a specific mitochondrial function for Aim32 was not assigned.

The thioredoxin fold of Aim32 is commonly found in oxidoreductases and chaperones (25). Proteins with a TLF domain may function as a thiol-based molecular switch by modulating disulfide bond formation in target proteins (26, 27, 28). Depending on the structure around the redox-active cysteine pair, the redox potential of thioredoxins can differ considerably. Moreover, mutagenesis of cysteine residues within the characteristic CXXC motif of thioredoxin proteins considerably changes the redox potential, modulating the properties of such proteins freely between reducing and oxidizing activities (29, 30, 31). Thus, Fe–S proteins are unique in that they have the potential of playing multiple roles in redox sensing and electron transfer within a particular system, and versatile in their adaptability to the redox status of the cell. Despite this, the exact biological function(s) of Aim32, and thioredoxin-like [2Fe-2S] ferredoxins in general, is not well understood.

In this study, we characterize the biological function of the TLF protein, Aim32, within the mitochondria. Using several complementary approaches, we first demonstrate that Aim32 is dually localized to the matrix and IMS of the mitochondria where it is essential for anaerobiosis. Aim32 has several binding partners within the IMS, inner membrane (IM), and the matrix. In the IMS, interactions with Erv1 and Osm1 may support a role in import, whereas additional interactions with translocon proteins including the TIM22 and TIM23 complexes suggest a role in protein stabilization or assembly of protein import complexes. Herein, we show that Aim32 may have a quality control function to maintain the assembly of protein complexes in which components may have thiols that are sensitive to damage.

## Results

### Aim32, Osm1, and Erv1 form a functional complex

Yeast must have other terminal electron acceptors in addition to cytochrome c (cyt c) and the Osm1/fumarate couple because Δ*cyc3*Δ*osm1* cells were viable under anaerobic conditions (12). Because Erv1 assembles in several distinct complexes, we sought to identify additional partner proteins of Erv1. The HISPC tag (termed CNAP for consecutive nondenaturing affinity purification) (32) was integrated in frame at the C terminus of Erv1 in plasmids that expressed ERV1 or the *erv1-12* allele from the endogenous promoter. The tagged proteins were designated as Erv1-HISPC and *erv1-12*-HISPC (Table S1). The plasmids were introduced into a strain deleted for *ERV1* by plasmid shuffling (10). Addition of the tag did not alter growth properties or Erv1 localization, supporting that Erv1 function was not impaired (data not shown). Preparative scale pull-downs were performed with the aforementioned strains and the final concentrated eluates were resolved by 10 to 15% SDS-PAGE. Compared with the negative control (WT + empty vector [Vec]), the proteins (highlighted by arrows) copurified strongly with *erv1-12*-HISPC extracts (Fig. S1). The bands were excised from the gel and digested with trypsin, and the proteins were identified by MS. Specifically, a new partner protein, Aim32, was enriched in the mitochondrial lysates from the *erv1*-*12*-HISPC yeast strain compared with the control, untagged WT strain (Fig. S1, and Table S2).

**Figure 1:**
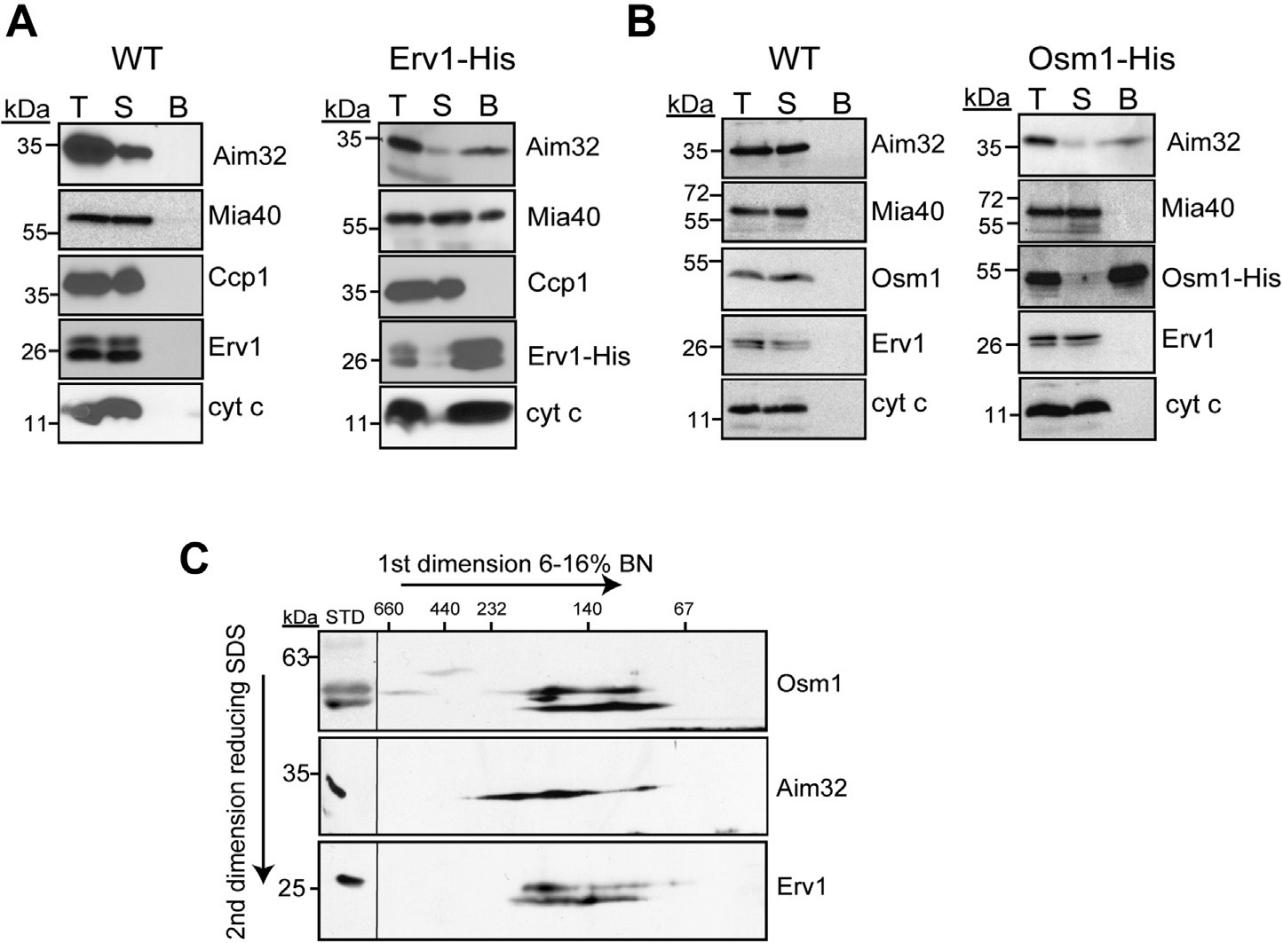
**Aim32 binds to Osm1 and Erv1.***A*, mitochondria from WT (*left panel*) or a strain expressing a C-terminal histidine-tagged Erv1 (Erv1-His) (*right panel*) were solubilized in 1% digitonin. As a control, 25 μg of the total extract (T) was withdrawn, and 500-μg lysate was incubated with Ni^2+^-agarose beads. The beads were washed, and bound protein (*B*) was eluted. 25 μg of the flow-through fraction (S) was also included. Samples were resolved by SDS-PAGE and analyzed by immunoblotting with specific antibodies against Aim32, Mia40, Ccp1, Erv1, and cyt *c*. *B*, as in *panel A*, except that mitochondria from a strain expressing a C-terminal histidine–tagged Osm1 (Osm1-His) was used. *C*, WT mitochondria were solubilized as in *panel A* and then separated in the first dimension on a 6 to 16% BN-PAGE gel, followed by reducing SDS-PAGE gel in the second dimension. Aim32, Osm1, and Erv1 were detected by immunoblotting. Aim32, altered inheritance of mitochondria 32; BN-PAGE, Blue Native PAGE; cyt c, cytochrome *c*.

We confirmed the interaction between Erv1 and Aim32 using a strain in which Erv1 was tagged with a C-terminal histidine tag (Erv1-His) (10). Similar to cyt *c* and Mia40, a fraction (∼10%) of Aim32 copurified with Erv1; as a control, the tested proteins were not detected in the untagged WT strain (Fig. 1*A*). Because we have identified Osm1 as a new partner protein of Erv1 (12), we performed a similar analysis to determine if Aim32 copurified with a C-terminal His-tagged Osm1 (Fig. 1*B*). We utilized the Osm1-His strain (12) and found that a fraction (∼5%) of Aim32 copurified with Osm1. Using two-dimensional (2D) gel analysis (Blue Native PAGE [BN-PAGE] followed by reducing SDS-PAGE), Aim32 was present in several complexes ranging between 70 and 230 kDa and comigrated with complexes that contained Erv1 (∼100–200 kDa) and Osm1 (∼100–200 kDa) (Fig. 1*C*). In sum, we demonstrate that a subset of Aim32 interacts with Erv1 and Osm1 in the IMS and migrates in similar-sized complexes.

### Aim32 is dual-localized to the IMS and matrix of the mitochondria

**Figure 2:**
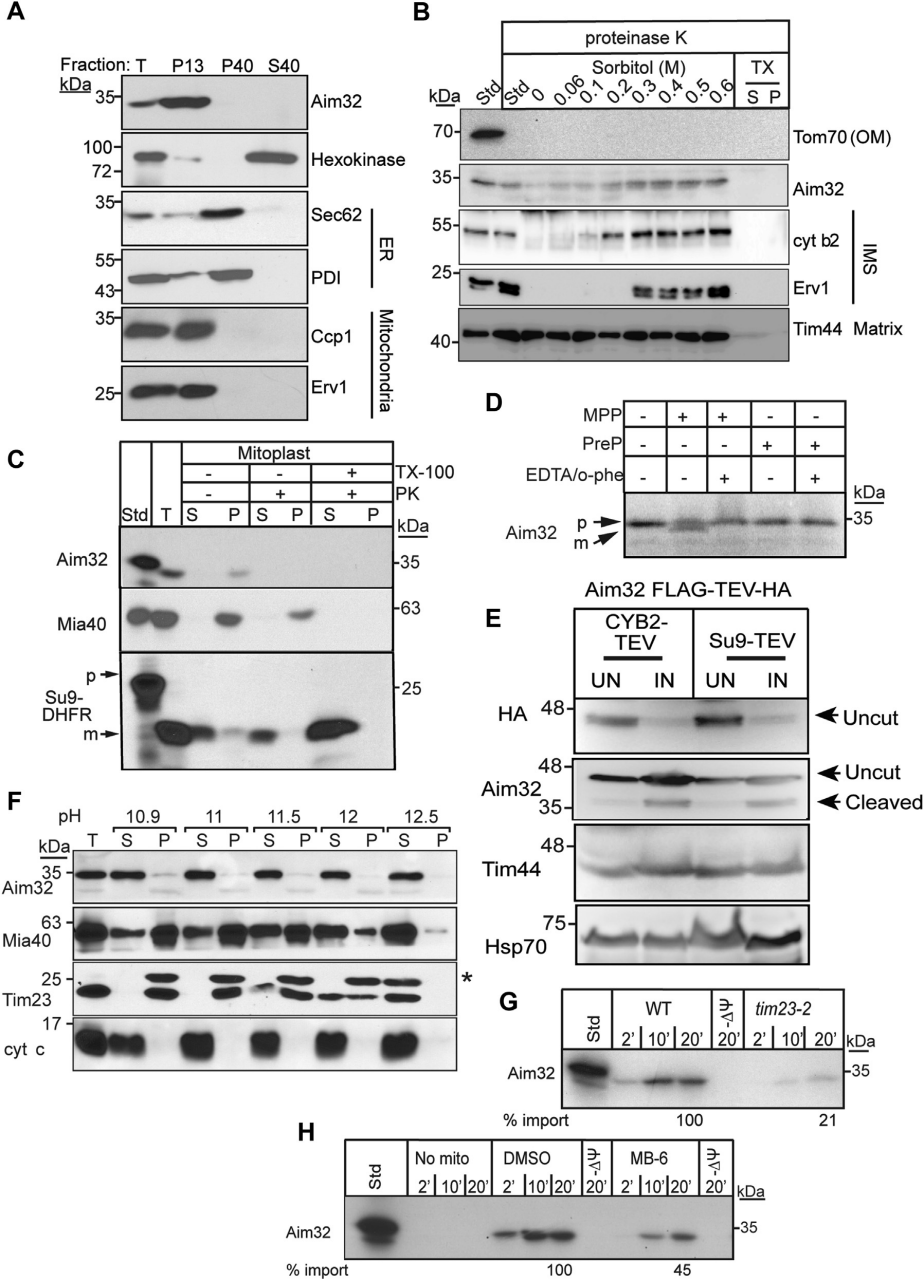
**Aim32 is a soluble protein of the mitochondrial IMS and matrix, and the import is facilitated by the TIM23 translocon.***A*, the WT strain was grown in YPEG at 30 °C and converted to spheroplasts. The total homogenate (T) was fractionated into mitochondria (P13), microsomes (P40), and cytoplasm (S40). An equal amount of each fraction was separated by SDS-PAGE and analyzed by immunoblot using the indicated antibodies. Markers include hexokinase (cytosol), Sec62 and PDI (ER), and Ccp1 and Erv1 (mitochondria). *B*, mitochondria were incubated in 20 mM Hepes-KOH, pH 7.4, 100 μg/ml proteinase K, and the indicated sorbitol concentrations at 4 °C for 30 min, followed by addition of 1 mM PMSF. After centrifugation, the pellet was analyzed by SDS-PAGE and immunoblotted with antibodies against the indicated proteins. *C*, import of radiolabeled Aim32 into WT mitochondria was followed by osmotic shock (final concentration 20 mM Hepes-KOH, pH 7.4, 100 μg/ml proteinase K [PK] and 0.06 M sorbitol, termed Mitoplast) as described in panel B. Su9-DHFR (matrix-targeted) and Mia40 (IMS-targeted) were used as controls. *D*, mitochondrial processing peptidase (MPP) cleavage assay was performed with the addition of 10 μg recombinant MPP to radiolabeled Aim32, and the samples were resolved on a 12% Tris-Tricine gel. As a negative control, 10 μg recombinant presequence protease (Cym1) was added to radiolabeled Aim32. 5 mM EDTA and 2 mM *o*-phenanthroline were added to inhibit activity of MPP. Samples were separated by SDS-PAGE and visualized using autoradiography. *E*, Δ*aim32* cells expressing Aim32-FLAG-TEV-HA were transformed with plasmids expressing matrix-localized Su9-TEV protease or IMS-localized CYB2 [1-220]-TEV protease. Cells were grown in minimal media supplemented with 2% galactose (IN) or 2% sucrose (UN) and harvested in the midlog phase. Whole-cell extracts were analyzed by immunoblotting against proteins Aim32, Tim44, Hsp70, and HA. *Arrows* indicate uncut and cleaved Aim32 FLAG-TEV-HA proteins. *F*, WT mitochondria were analyzed by alkali extraction using 0.1 M carbonate at the indicated pH. Equal amounts of the pellet (P) and TCA-precipitated supernatant (S) fractions from 50 μg mitochondria were resolved by SDS-PAGE and immunoblotted for the indicated mitochondrial markers. The *asterisk* indicates nonspecific band. *G*, radiolabeled Aim32 was imported into WT and *tim23-2* mutant mitochondria in the indicated time course. Nonimported precursor was removed by protease treatment, and the imported Aim32 was analyzed by SDS-PAGE and autoradiography. “-Ψ” indicates import when the membrane potential was dissipated with 1 μM valinomycin. A 10% standard (Std) from the translation reaction was included. Import reactions were quantitated using ImageJ software; 100% was set as the amount of precursor that imported into WT mitochondria at the endpoint of the time course. A representative gel is shown (*n* = 3). *H*, as in *panel G*, Aim32 was imported into WT mitochondria in the presence and absence of a ΔΨ and 25 μM of MitoBLoCK-6 (42) or a vehicle control [1% dimethyl sulfoxide (DMSO)]. Aim32, altered inheritance of mitochondria 32; ER, endoplasmic reticulum; IMS, intermembrane space; IN, induced; m, mature; MIA, mitochondrial intermembrane space assembly; OM, outer membrane; p, precursor; UN, uninduced.

To confirm a function in mitochondria, we localized Aim32 (Fig. 2*A*). Lysates from spheroplasts were subjected to differential centrifugation, and fractions from the 13K pellet (mitochondria) and the 40K supernatant (cytosol) and 40K pellet (microsomes) were separated by SDS-PAGE. Immunoblotting with appropriate markers, Erv1 and Ccp1 for mitochondria, hexokinase for cytosol, and protein disulfide isomerase and Sec62 for the ER, verified integrity of the fractionation. Aim32 purified with the mitochondrial fraction.

The submitochondrial localization of Aim32 was determined using osmotic shock in the presence of proteinase K (Fig. 2*B*). Outer member marker Tom70 was degraded immediately, whereas matrix marker Tim44 was inaccessible to protease, even in low sorbitol concentration. IMS markers cyt *b*_2_ and Erv1 were degraded as the sorbitol concentration decreased. Aim32 showed a similar degradation pattern to the IMS markers. As a control, the addition of detergent resulted in degradation of all proteins. Of note, a small fraction of Aim32 remained protease resistant at 0 to 0.1 M sorbitol; this may indicate that osmotic shock did not lyse the mitochondrial outer member or a fraction of Aim32 may be localized to the matrix.

We used an alternative strategy with *in vitro* import of radiolabeled Aim32 followed by osmotic shock to confirm mitochondrial localization (Fig. 2*C*). Imported Mia40 and Aim32 were cleaved by proteinase K, but matrix-localized Su9-DHFR was protected from protease. The addition of detergent also resulted in degradation of Aim32 and Mia40; DHFR was not efficiently degraded because it was tightly folded (12). The imported form of Aim32 was slightly smaller than the translated standard, which suggests that the N-terminal targeting sequence may be cleaved (Fig. 2*C*). To confirm this, using recombinant matrix processing peptidase (MPP) or the presequencing processing peptidase (Cym1), cleavage of radiolabeled Aim32 was tested (33). Based on a putative cleavage site at the 13th amino acid (predicted using MitoFates (34)), MPP likely cleaved Aim32 at the N terminus (Fig. 2*D*), resulting in a ∼1-kDa shift. In contrast, MPP that was inactivated by the addition of EDTA and *o*-phenanthroline (35) as well as Cym1 failed to cleave Aim32. MPP activity was confirmed by cleavage of the Su9-targeting sequence in Su9-DHFR (Fig. S2*A*). Thus, during mitochondrial protein import, the N terminus of Aim32 enters the matrix for processing by MPP.

Because previous studies localized Aim32 to the matrix (24, 36), which contrasts with our sorbitol gradient assays and IMS-based interactions with Erv1 and Osm1, we utilized the tobacco etch virus (TEV) protease cleavage assay to determine the mitochondrial localization of Aim32 *in vivo* (37, 38). The TEV protease, under control of an inducible promoter, was targeted to the IMS (CYB2-TEV) or the matrix (Su9-TEV). A construct that contains Aim32 with a 3X-FLAG tag, TEV cleavage site, and HA tag (Aim32 FLAG-TEV-HA) was cotransformed, and processing of Aim32 was examined (Fig. 2*E*). When the TEV protease was induced in both the IMS and matrix locations, the HA tag from Aim32 was cleaved and an antibody against Aim32 confirmed the presence of this Aim32 form, which corresponds to removal of the ∼5-kDa HA tag. Although the processing of Aim32 FLAG-TEV-HA was not complete, additional control experiments indicated that this cleavage was specific to CYB2-TEV and Su9-TEV. In particular, a similarly tagged construct expressing Nuc1 that localizes exclusively to the IMS (39) was processed only when the IMS localized TEV protease (CYB2-TEV) was induced (Fig. S2*B*). Such incomplete processing by the TEV proteases has been reported by Kondo-Okamoto *et al*. (37) for Mmm1p. In another set of control experiments, the processing of the construct expressing Sod2 with a 3X-FLAG tag, TEV cleavage site, and HA tag (Sod2 FLAG-TEV-HA) that is matrix-localized (data not shown) was examined. Sod2 FLAG-TEV-HA was processed successfully by the coexpression of the matrix-localized TEV protease (Su9-TEV) but not the IMS-localized CYB2-TEV protease (Fig. S2*C*). A minor species of cleaved Sod2 FLAG-TEV-HA was visible in all conditions. However, coexpression of the Su9-TEV resulted in the bulk of the protein being cleaved, reflected by the size shift that corresponds to the removal of the ∼5-kDa HA tag (Fig. S2*C*).

Aim32 association with the membrane was tested using carbonate extraction over the pH range 10.5 to 12.5 (Fig. 2*F*) (40). In control reactions, integral membrane protein Tim23 remained in the pellet until pH 12, whereas soluble cyt *c* was present in the supernatant over the entire pH range. Mia40, which is anchored by a single transmembrane domain, showed an intermediate pattern switching from the pellet to the supernatant at pH 12. Aim32 remained in the supernatant over the entire pH range, indicating that Aim32 is a soluble protein. In sum, Aim32 is a soluble protein that localizes to the mitochondrial IMS and matrix.

Additional characterization of the Aim32 import pathway revealed that Aim32 import required a membrane potential (Δψ) for translocation, and this import was reduced by 80% in the *tim23-2* mutant mitochondria ((41) and Fig. 2*G*). Aim32 import in the presence of the Erv1-specific inhibitor MitoBloCK-6 (42) was also reduced by 55% (Fig. 2*H*). Thus, Aim32 translocation requires the TIM23 translocon for import into the mitochondrial matrix, and the MIA pathway may be required for partitioning Aim32 to the IMS or for modification of redox properties.

### Aim32 is structurally similar to TLF proteins

Multiple protein sequence alignment using PROMALS (43) shows that the C-terminal portion of Aim32 sequence aligns well with the sucrase/ferredoxin-like family proteins from *Arabidopsis thaliana* (UniProt identifier, Q9FG75) and *Aspergillus fumigatus* (UniProt identifier, Q4WDI8). Secondary structure predictions revealed that the C-terminal region of Aim32 (∼100 amino acids) has an overall α-helix/β-strand architecture, which is a variation of the classical thioredoxin fold (Fig. S3*A*). Furthermore, the C-terminal TLF domain contains a CX_8_C and HX_3_H motifs and a key tryptophan residue that is conserved among other known ferredoxin proteins (Fig. S3*B*). Our analyses are in agreement with studies published by Stegmaier *et al*. (24) that experimentally show that Aim32 coordinates a [2Fe-2S] cluster. Thus, Aim32 is a bona fide [2Fe-2S] protein, and the CX_8_CX_n_HX_3_H motif within Aim32 most likely constitutes its active site.

### Conserved residues within the CX_8_CX_n_HX_3_H motif of Aim32 are important for function

**Figure 3:**
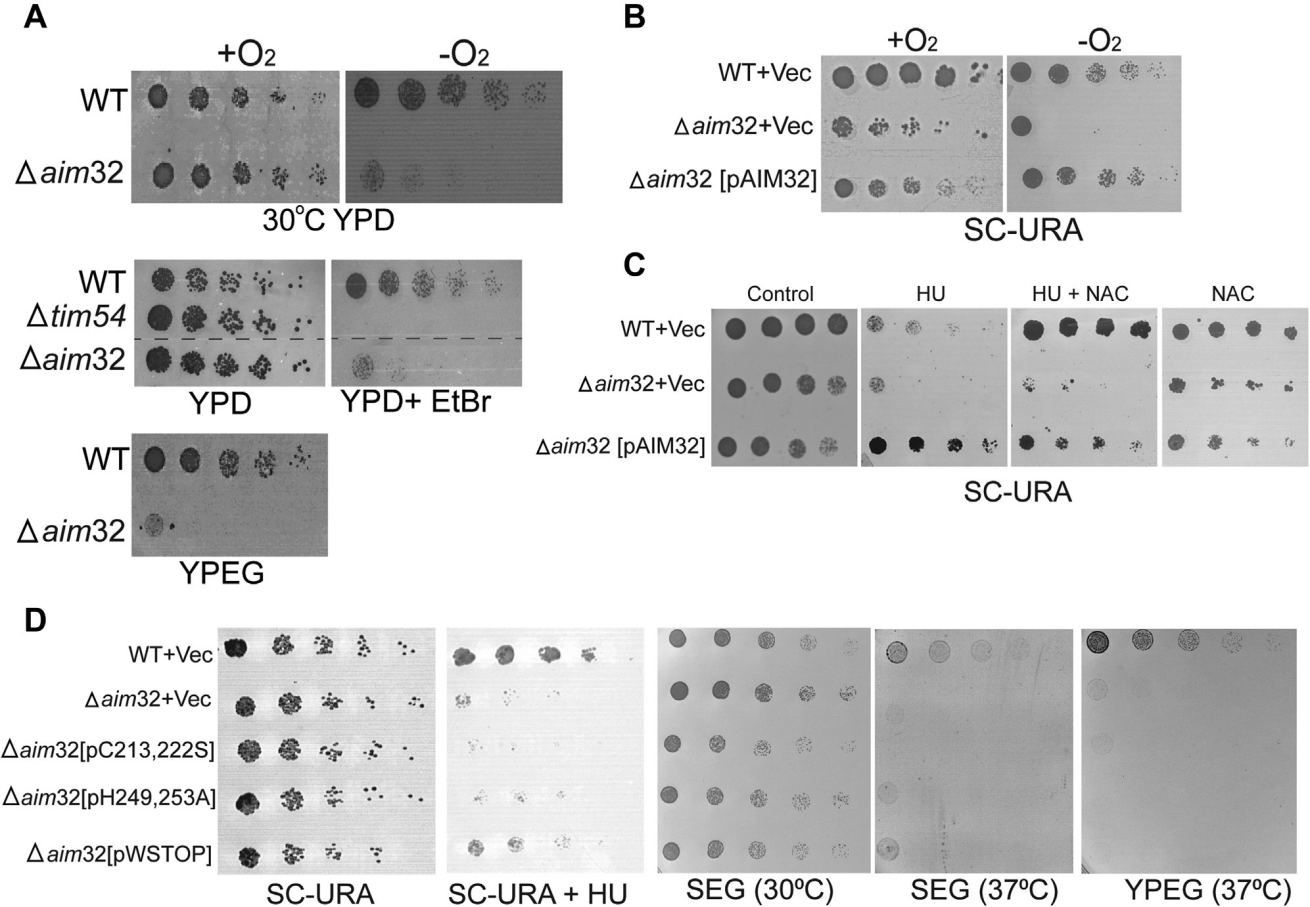
**Conserved residues within the TLF domain of Aim32 are essential for mitochondrial respiration.***A*, growth analysis of Δ*aim32* and the parental (WT) strains in aerobic (+O_2_) and anaerobic (−O_2_) conditions on rich glucose (YPD) and ethanol/glycerol (YPEG) media was analyzed by serial 5-fold dilution of liquid cultures grown to the midlog phase. Ethidium bromide (EtBr) treatment removed the mitochondrial DNA. The *tim54-1* mutant (petite-negative) was included as a control. Plates were incubated at 30 °C (*B*) WT, and *Δaim32* strains were transformed with an empty plasmid (+Vec) or plasmid expressing Aim32 protein (pAIM32). Serial dilutions as in *panel A* were spotted onto selective minimal glucose media lacking uracil (SC-URA) and growth observed after 2 to 3 days at 30 °C. *C*, as in *panel B*, with the addition of 100 mM hydroxyurea (HU) or 150 mM N-acetyl-cysteine (NAC). Growth was observed after 3 days at 30 °C. *D*, *aim32* mutants were tested as in *panel C* for sensitivity to hydroxyurea or different carbon sources, on minimal ethanol/glycerol (SEG) lacking uracil or rich ethanol/glycerol (YPEG) media at 30 °C and 37 °C. Photographs were taken after 2 to 3 days of incubation. Data shown are representative of three independent experiments. Aim32, altered inheritance of mitochondria 32; TLF, thioredoxin-like ferredoxin.

The requirement for Aim32 under different growth conditions was tested with a strain deleted for *AIM32* (*Δaim32*). *AIM32* is not an essential gene but is required for growth on rich or minimal glucose medium under anaerobic conditions and on respiratory media at 37 °C (Fig. 3, *A* and *B*). When rich glucose media was supplemented with ethidium bromide (EtBr), which causes loss of mitochondrial DNA (referred to as ρ^0^; (44)), the *Δaim32* strain and control *Δtim54* strain were not viable (Fig. 3*A*). Growth in the presence of hydroxyurea (HU) and reductant N-acetylcysteine was also tested because deletion of the *AIM32* homolog, *APD1*, results in HU sensitivity (24, 45). The Δ*aim32* strain was sensitive to HU, and the addition of N-acetylcysteine did not restore growth (Fig. 3*C*). Furthermore, the *Δaim32* strain displayed increased growth sensitivity in the presence of an uncoupler (Fig. S4*A*). However, growth defects observed under anaerobic conditions and in the presence of HU were rescued by subsequent expression of plasmid-borne *AIM32* (Figure 3, *B* and *C* and Fig. S4*D*). No change in mitochondrial network morphology was observed between the *Δaim32* and parental WT strain (Fig. S4*C*). Thus, the *Δaim32* strain has a petite-negative phenotype, and Aim32 is required for respiration at elevated temperatures, and against HU-induced stress. To address the importance of the conserved residues in the CX_8_CX_n_HX_3_H motif as well as the conserved tryptophan (Fig. S3*B*), strains that contained mutations in these residues were constructed (termed pC213, 222S; pH249, 253A; pWStop that terminates at lysine 279 and eliminates the conserved tryptophan and additional 33 C-terminal amino acids). Plasmids expressing WT and *aim32* mutants were transformed into *Δaim32* cells; Aim32 expression was verified by immunoblotting (Fig. S4*E*). The strains were tested for growth in the presence of HU and on respiratory media (Fig. 3*D*); all mutants showed reduced growth on HU and respiratory media at 37 °C. As in Figure S4*A*, the Aim32 mutants also showed decreased growth in the presence of mitochondrial uncoupler (Fig. S4*B*). Thus, the cysteine and histidine residues that coordinate the [2Fe-2S] cluster (24) and the terminal tryptophan residue are important for Aim32 function.

**Figure 4:**
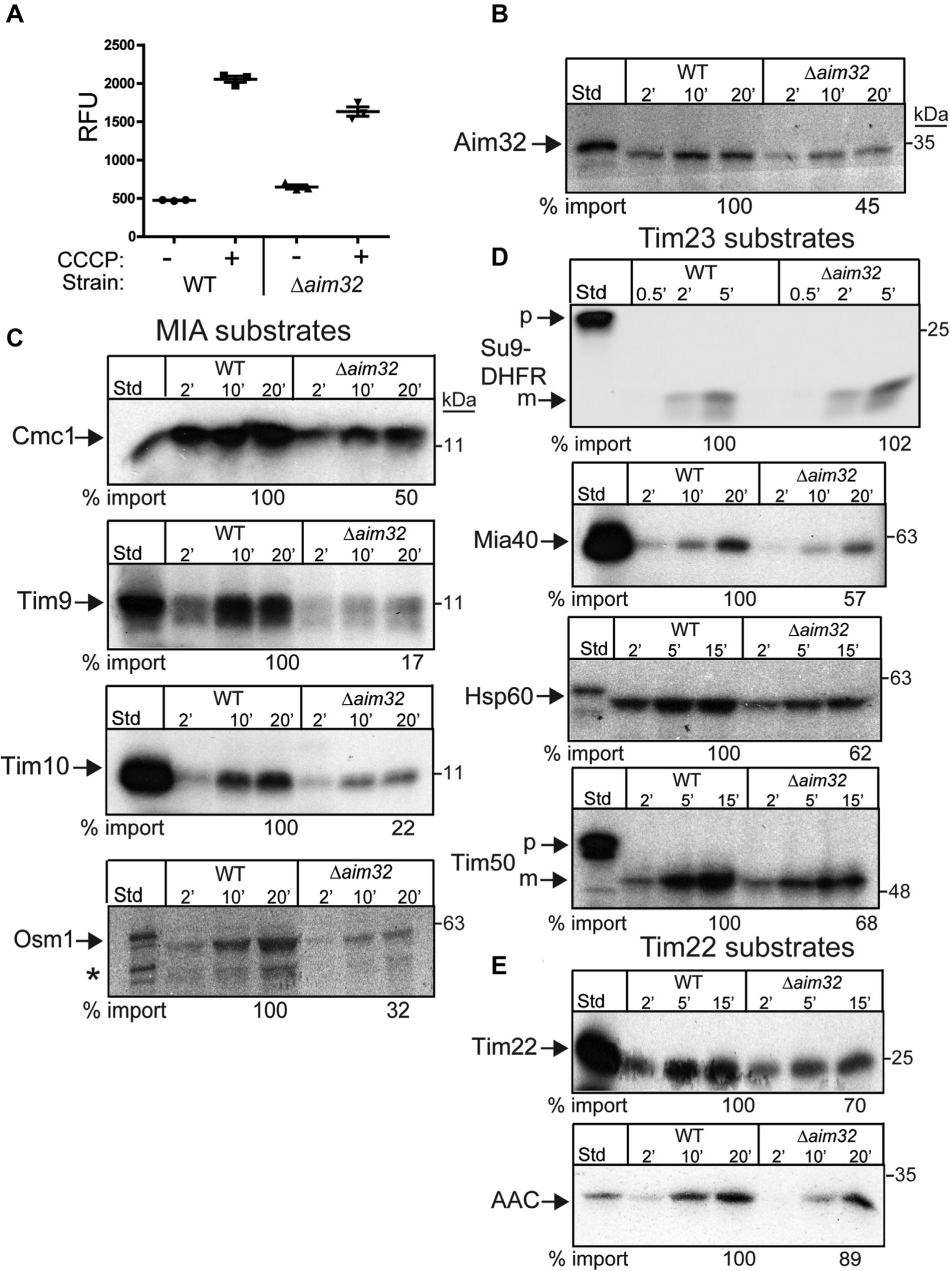
**Cells lacking functional AIM32 are defective in import of diverse mitochondrial precursors.***A*, membrane potential (ΔΨ) measurements of purified mitochondria from WT and *Δaim32* strains were performed with DiSC_3_ (5) and measured with a FlexStation plate reader (Molecular Devices). Coupled mitochondria sequestered and quenched the dye fluorescence; collapse of the ΔΨ was achieved with CCCP addition. *B*, radiolabeled Aim32 was imported into WT, and *Δaim32* mitochondria as in Figure 2*G*. A representative gel is shown (*n* = 3). *C*, as in *panel B*, representative MIA precursors (Cmc1, Tim9, Tim10, and Osm1) were imported. *D*, as in *panel B*, TIM23 substrates (Su9-DHFR, Mia40, Hsp60, and Tim50) were imported. *E*, as in *panel B*, import of TIM22 substrates (Tim22 and ACC) was tested. Samples were subjected to carbonate extraction after protease treatment. The *asterisk* denotes nonspecific band. Aim32, altered inheritance of mitochondria 32; CCCP, carbonyl cyanide m-chlorophenyl hydrazone; DiSC3 (5), 3, 3′-dipropylthiadicarbocyanine iodide; MIA, mitochondrial intermembrane space assembly; m, mature; p, precursor.

### Aim32 is required for assembly of the import complexes

**Figure 5:**
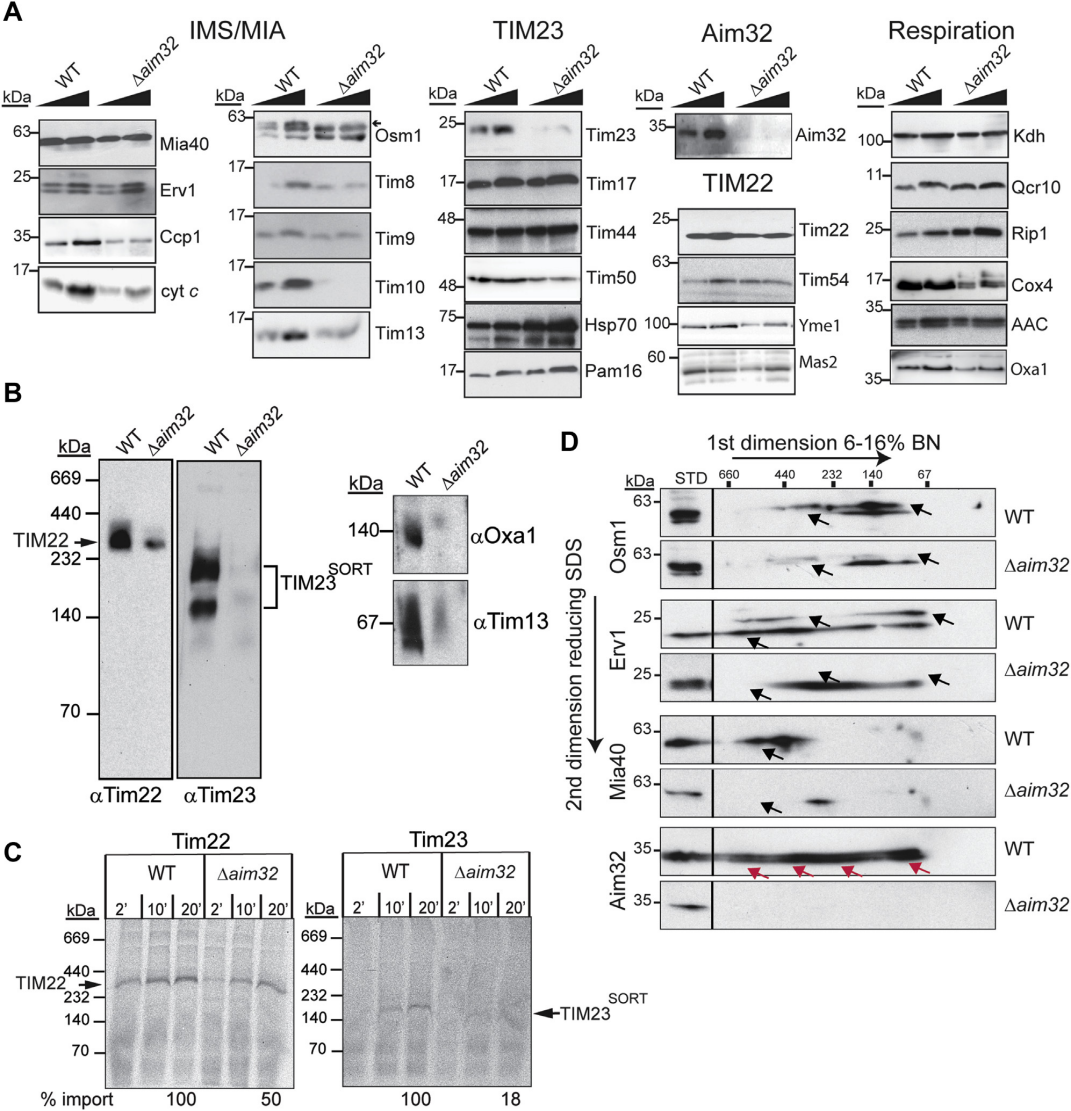
**Aim32 functions in stabilization of several protein complexes.***A*, a systematic analysis of steady-state levels of mitochondrial proteins (50 and 100 μg) from the parent (WT) or Δ*aim32* strain (grown at 30 °C in rich glucose media) was performed by immunoblot with antibodies against the indicated mitochondrial proteins. Proteins were organized by substrates of the MIA, TIM23, and TIM22 pathways and respiratory proteins. *B*, 75 μg mitochondria from the parent (WT) or Δ*aim32* strain were solubilized in 1% (wt/vol) digitonin and subjected to BN-PAGE (6–16% acrylamide) to detect TIM22, TIM23, Oxa1, and small TIM complexes. *C*, radiolabeled Tim22 or Tim23 was imported into WT or Δ*aim32* mitochondria in a time course and subjected to BN-PAGE and digital autoradiography. Import reactions were quantitated using ImageJ software; 100% was set as the amount of precursor that imported into WT mitochondria at the endpoint of the time course. A representative gel is shown (*n* = 3). *D*, as in *panel B*, except that BN-PAGE was followed by a reducing SDS-PAGE in the second dimension (2D), and the indicated antibodies were used. *Red arrows* highlight Aim32-containing complexes identified in WT mitochondria. *Black arrows* indicate protein-containing complexes in the WT mitochondria that are absent in the Δ*aim32* mitochondria. Note that Erv1 and Osm1 typically run as a doublet. Aim32, altered inheritance of mitochondria 32; BN-PAGE, Blue Native PAGE; MIA, mitochondrial intermembrane space assembly.

Because Aim32 interacts with Erv1, we performed import studies with mitochondria from WT and *Δaim32* cells. The Δψ of isolated mitochondria was measured using the fluorescent dye 3, 3′-dipropylthiadicarbocyanine iodide [DiSC3 (5)] (46). Mitochondria from the *Δaim32* strain had a decreased Δψ of ∼20% (Fig. 4*A*). Aim32 seemed to depend on itself for translocation because import was compromised by 55% in *Δaim32* mitochondria (Fig. 4*B*). The import of MIA substrates Tim9 and Tim10 was decreased by ∼80%, whereas the import of Cmc1 was reduced by ∼50% (Fig. 4*C*). However, a modest reduction for a subset of TIM23 substrates (Tim50, Hsp60, and Mia40) and TIM22 substrates (Tim22 and AAC) in import was noted, whereas import of Su9-DHFR was not compromised (Fig. 4, *D* and *E*). Finally, import of Osm1 into *Δaim32* mitochondria was strongly decreased. Thus, mitochondria that lack Aim32 are generally compromised in protein import, particularly for proteins that use the MIA pathway. To complement the import studies, the steady-state level of mitochondrial proteins was determined by immunoblot analysis (Fig. 5*A*). Mitochondria were purified from the WT and Δ*aim32* strains grown in rich glucose media at 30 °C to the midlog phase. In agreement with reduced import of the MIA substrates, the abundance of the small Tim proteins was diminished in the Δ*aim32* strain. With the exception of Tim23 and Oxa1, the steady state level of additional mitochondrial proteins in the TIM23, TIM22, and MIA and respiratory complexes was not markedly reduced. We followed with BN gel analysis to assess complex assembly. The TIM22, TIM23, and small Tim complexes were markedly decreased in mitochondria from the Δ*aim32* strain (Fig. 5*B*). Tim23 assembly into the TIM23^SORT^ complex was particularly defective. In addition, Oxa1 assembly in a large complex was decreased (Fig. 5*B*). Similar defects in complex assembly were observed when Tim22 and Tim23 import and complex assembly was analyzed by BN gels (Fig. 5*C*). With 2D BN/SDS-PAGE analysis, complexes with Osm1, Erv1, and Mia40 were monitored in WT and *Δaim32* mitochondria. Four Aim32 complexes of ∼140 to 500 kDa are highlighted with red arrows (Fig. 5*D*). In *Δaim32* mitochondria, assembly of Erv1, Mia40, and Osm1 complexes was markedly altered (indicated by black arrows in Fig. 5*D*), although the steady state levels of these proteins were not decreased. Thus, Aim32 seems to be required for correct assembly/stability of numerous import complexes.

### Aim32 has multiple binding partners

**Figure 6:**
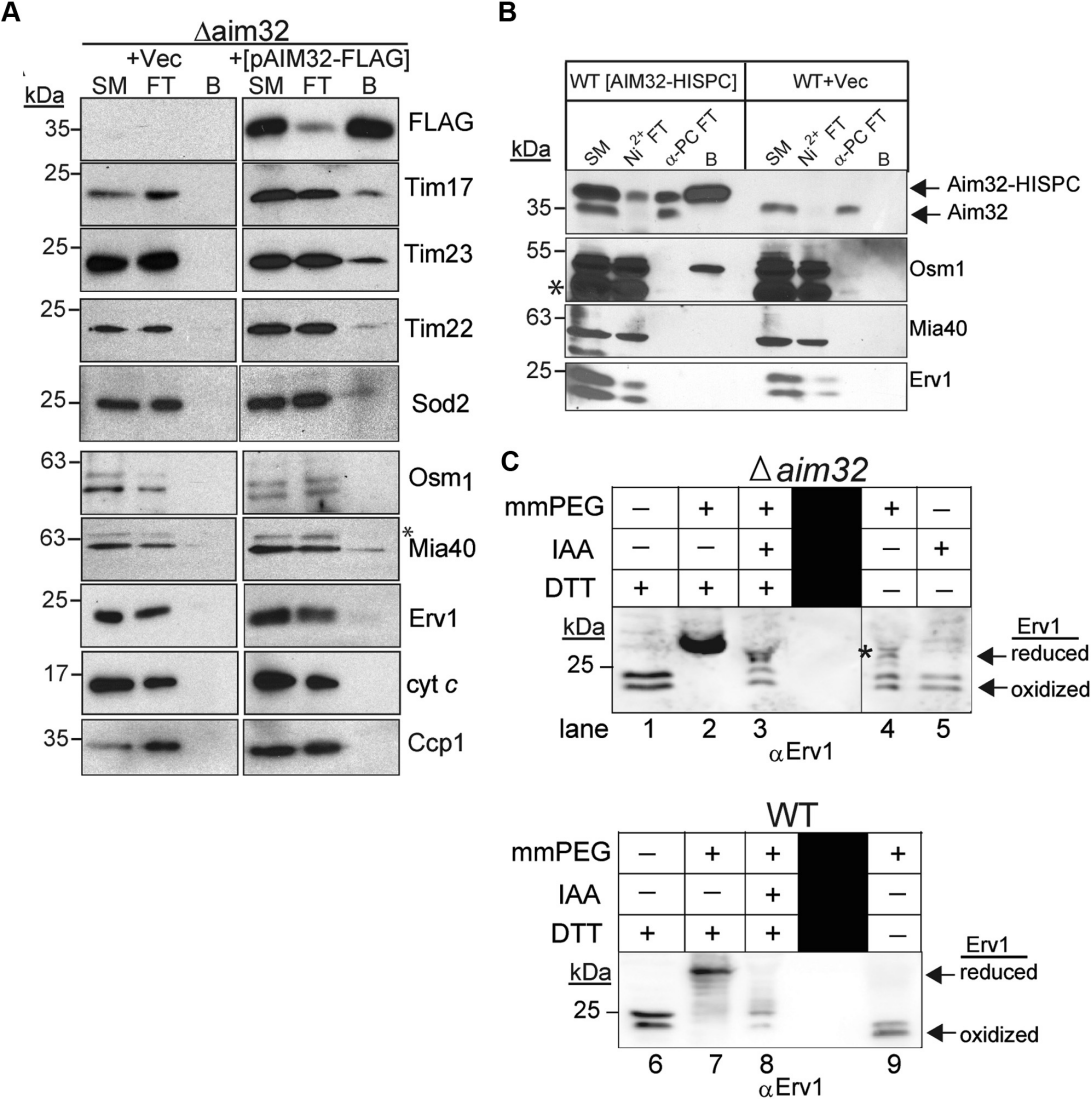
**Aim32 interacts with key proteins of the MIA, TIM23, and TIM22 pathways.***A*, a FLAG tag was appended to the C terminus of Aim32 (Aim32-FLAG) and transformed into *Δaim32*. Pull-down assays with FLAG-resin were performed as in Figure 1*A* with the indicated antibodies. An *asterisk* indicates nonspecific bands detected by the antibody. *B*, a consecutive-affinity tag (termed CNAP for purification over Ni^2+^-resin followed by anti-Protein C affinity (α-PC) resin) was placed on the C terminus of Aim32. As in Figure 1*A*, the lysate was incubated with Ni^2+^-agarose, eluted with 300 mM imidazole, and then incubated with the anti-protein C resin. After washing, the bound material was eluted and the indicated antibodies were used. Controls include the “flow-through” material from the Ni^2+^-agarose (Ni^+2^-FT) and the anti-protein C resin (α-PCFT). *C*, analysis of the Erv1 oxidation state by thiol-trapping assay. Isolated mitochondria from *Δaim32* (lanes 1–5) and WT (lanes 6–9) were resuspended in the sample buffer with dithiothreitol (DTT, lanes 1 and 6), iodoacetamide (IAA, lane 5), and methyl-PEG_24_-maleimide (mmPEG, lanes 4 and 9), or pretreated with IAA to block free cysteine residues (lanes 3 and 8), and disulfide bonds subsequently reduced by DTT, and finally reacted with mmPEG (lanes 2, 3, 7, and 8). Samples were analyzed by SDS-PAGE and immunoblotting with the anti-Erv1 antibody. The *asterisk* indicates reduced Erv1 species. Aim32, altered inheritance of mitochondria 32; MIA, mitochondrial intermembrane space assembly.

Considering that import of diverse precursors and assembly of various import complexes is impaired in cells that lack Aim32, we used MS to identify potential Aim32-interacting proteins. Mitochondrial lysates from *Δaim32* strains expressing a C-terminal FLAG-tagged Aim32 ([pAIM32-FLAG], expression controlled from the GPD promoter) or an Vec were purified with FLAG beads and, after washing, subjected to MS. Several key components of the TIM23 translocon (Tim17, Tim23, and Tim50) and Mia40 were identified as potential binding partners (Table 1). To validate these interactions, specific copurifications were performed (Fig. 6*A*). A fraction (∼10%) of Tim17, Tim23, Tim22, Tim50, and Sod2 copurified with Aim32-FLAG. Reciprocal pull-downs were also performed with His-tagged Tim17 and Tim23 strains and Aim32 copurified with Tim17 and Tim23 (Fig. S5). A small fraction of Mia40 also copurified with FLAG-tagged Aim32, but Erv1 and Osm1 were not detected (Fig. 6*A*). To determine if the tag might impact interactions, we placed the HisPC tag on the C terminus of Aim32. Tandem affinity purifications performed with the Aim32-HisPC strain showed Osm1 copurifying with Aim32, but Erv1 and Mia40 were not detected (Fig. 6*B*). In sum, Aim32 seems to bind to several proteins, particularly import components, but the stability of interaction may be impacted by the presence or placement of a tag. Thus, Aim32 interactions may be transient with partner proteins.

**Table 1:** MS data containing a list of identified proteins, spectral counts, and sequence coverage from the Δ*aim32* [pAIM32-FLAG] and Δ*aim32* + Vec runs at a false-positive rate of less than 1%

Protein	Accession No.	Sequence count Δ*aim32* [pAIM32-FLAG]-IP	Spectral count Δ*aim32* [pAIM32-FLAG]-IP	Sequence count Δ*aim32* + Vec-IP	Spectral count Δ*aim32* + Vec-IP	Sequence coverage % Δ*aim32* [pAIM32-FLAG]-IP	Sequence coverage % Δ*aim32* + Vec IP
Tim23	YNR017W	6	8	0	0	50.5%	0
Tim17	YJL143W	2	2	0	0	21.5%	0
Sod2	YHR008C	8	15	5	6	53.2%	36.6%
Mia40	YKL195W	14	19	5	5	44.2%	16.10%

Because Aim32 was identified as an Erv1-binding partner, we investigated the redox status of Erv1 in mitochondria from WT and *Δaim32* strains using thiol trapping with methyl-PEG-24-maleimide (mmPEG) (Fig. 6*C*) (47). When presented with reduced substrates in *in vitro* reconstituted systems, the two shuttle cysteine residues as well as the active site cysteines are crucial for Erv1 oxidase activity (48). In control reactions in which Erv1 was treated with reductant DTT at 95 °C (Fig. 6*C*, lanes 2 and 7), six mmPEG molecules bound to Erv1, resulting in a mass shift of ∼7 kDa compared with treatment with DTT alone (lanes 1 and 6). In other control reactions, treatment with either iodoacetamide (IAA) or mmPEG alone (lanes 5 and 9) did not result in any mobility shifts. Pretreatment with IAA, followed by disulfide reduction with DTT and subsequent mmPEG modification (Fig. 6*C*, lane 3 and 8), resulted in a Erv1 species that migrated more slowly than reactions in lanes 2 and 7, thereby indicating partial oxidation of Erv1 by mmPEG. In WT mitochondria, Erv1 is present in an oxidized state (Fig. 6*C*, lane 9 mmPEG addition), whereas a subset of Erv1 remained reduced (marked with an asterisk, lane 4) in Δ*aim32* cells. Thus, thiol-trapping analysis suggests that Aim32 influences the redox status of Erv1 under aerobic conditions.

### Aim32 is required for maintaining the global redox status

**Figure 7:**
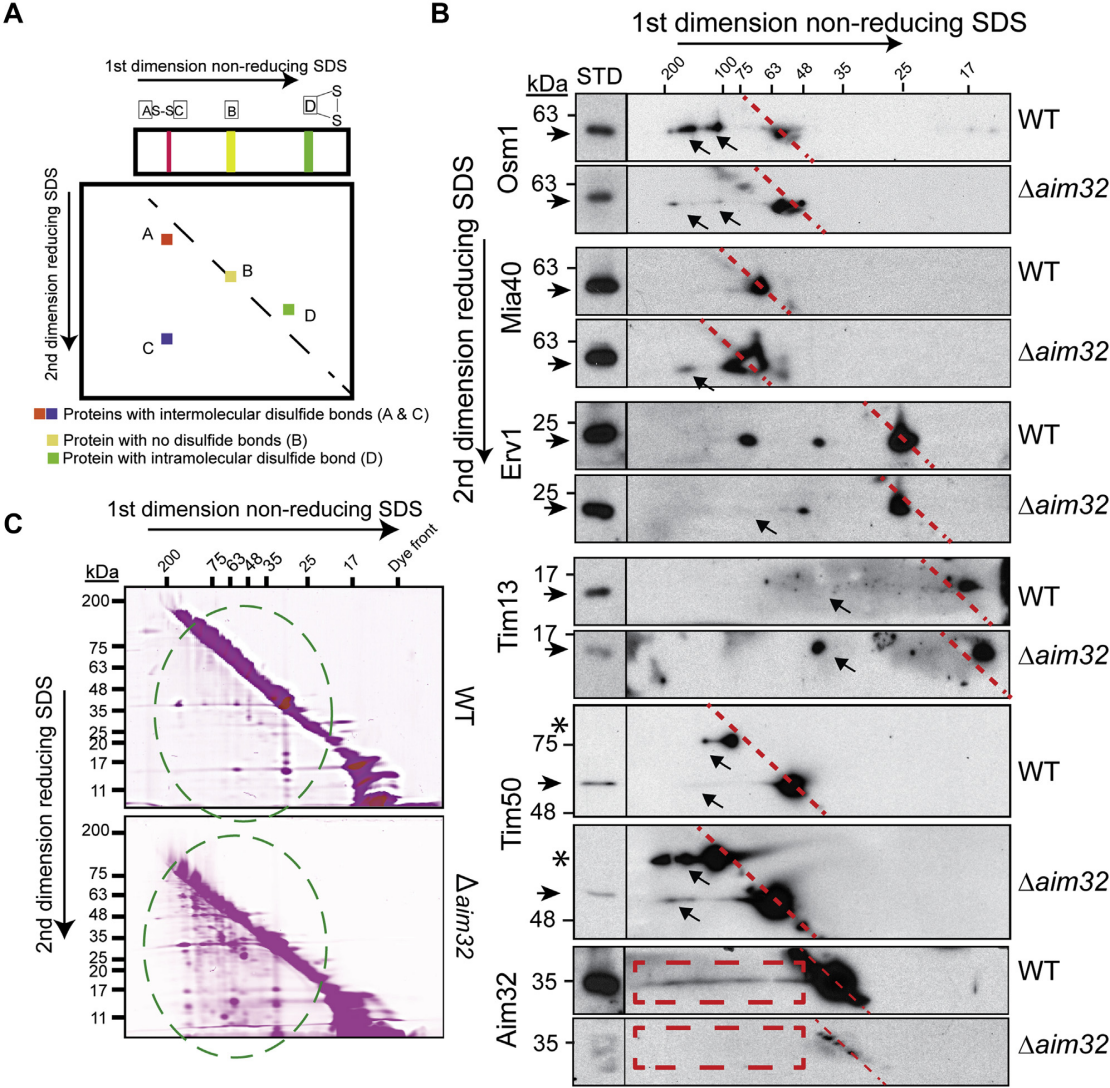
**Aim32 forms disulfide bonds with diverse mitochondrial proteins and alters the disulfide proteome.***A*, a general scheme for the 2D gel electrophoresis (2DGE), wherein samples were first separated under nonreducing conditions followed by reductant treatment in the second dimension. *B*, as in *panel A*, 2DGE was performed on mitochondrial lysates from WT and *Δaim32* strains and analyzed by immunoblotting. *Dashed red line* indicates the position of the diagonal. *Red boxes* refer to Aim32-specific heterodimers with other mitochondrial proteins in WT mitochondria. *Arrows* specify places where the disulfide linkages differ in WT vs *Δaim32* mitochondria. *C*, the general redox status of thiols in the mitochondria was assessed. As in *panel B*, except that 100-μg mitochondrial extract was pretreated with iodoacetamide labeled with rhodamine (IAA-rhodamine) to block free thiols followed by reduction in-gel with 1% β-mercaptoethanol. Gels were visualized using the Sapphire Biomolecular Imaging system. Representative gels are shown (*n* = 3). *Green dashed circle* indicates increased rhodamine-labeled proteins running under the diagonal in the Δ*aim32* mitochondrial extracts. *Asterisk* indicates higher molecular weight Tim50 species. Aim32, altered inheritance of mitochondria 32.

We postulated that analogous to thioredoxin proteins, Aim32 may function in maintaining the global redox status of the mitochondrial proteome. Two-dimensional diagonal gel electrophoresis (2D-DGE) in which the first dimension was nonreducing followed by a reduction in the second dimension was used (scheme in Fig. 7*A*). The redox status of specific proteins, previously found to interact with Aim32, was investigated by immunoblot analysis. Osm1, Mia40, Erv1, Tim13, and Tim50 had altered disulfide linkages (as highlighted by arrows in Fig. 7*B*) in the *Δaim32* strain. A fraction of Aim32 migrated below the diagonal (indicated by dashed box), indicating Aim32 seems to form disulfide linkages with several proteins under normal physiological conditions.

In a second approach, we probed the thiol proteome with IAA-labeled rhodamine (IAA-rhodamine), based on Hill *et al*. (49). Free thiols in mitochondrial lysates were reacted by irreversible alkylation with IAA-rhodamine and separated by a 2D-DGE as in Figure 7*B*. Increased IAA-rhodamine labeling of proteins with nonnative disulfide bonds was observed in the Δ*aim32* mitochondria (Fig. 7*C*, marked by dashed green circle). Thus, Aim32 plays a role in maintaining the global redox status.

### Conserved cysteine residues in the TLF domain of Aim32 are critical for function

**Figure 8:**
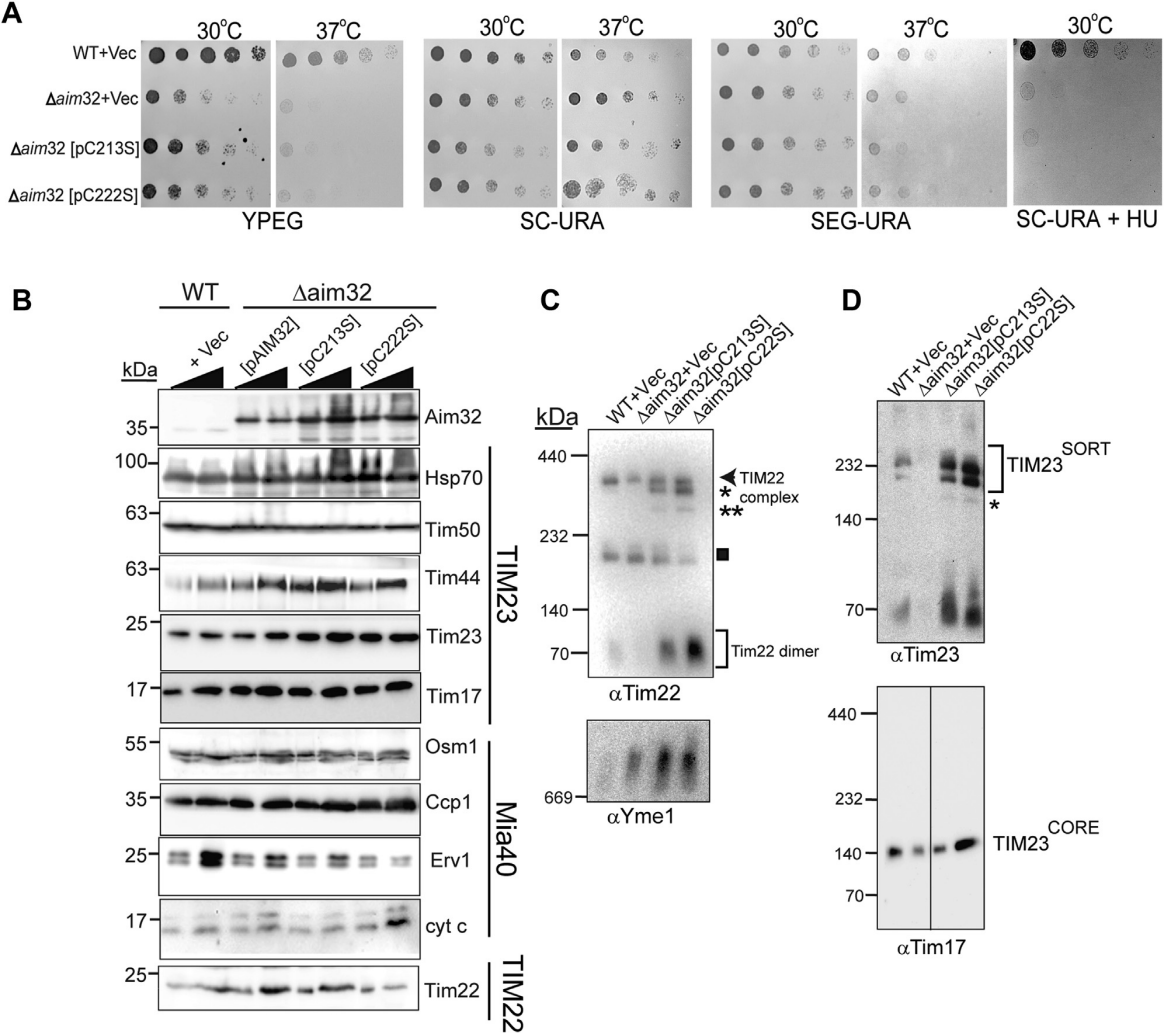
**Conserved cysteine residues of Aim32 are important for assembly/stabilization of the TIM22 and TIM23 complexes.***A*, growth analysis of the indicated yeast strains after 2 to 3 days of incubation at 30 °C and 37 °C on different media as in Figure 3. Strains include WT and *Δaim32* transformed with a plasmid that contains *AIM32* with a single cysteine mutation (C213S or C222S) and a C-terminal FLAG tag or the empty vector (Vec). Data shown are representative of three independent experiments. *B*, as in Figure 5*A*, levels of mitochondrial proteins were analyzed by immunoblotting with antibodies against the indicated proteins. Strains are from *panel A* and also include AIM32 with a C-terminal FLAG tag (pAIM32). *C*, as in Figure 5*B*, 75-μg mitochondria from the indicated strains were solubilized in 1% (wt/vol) digitonin and subjected to blue native (BN)-PAGE (4–16% acrylamide) to detect TIM22, and *D*. TIM23^SORT^, and the TIM23^CORE^ complexes. For the Yme1 complex, a 3 to 12% acrylamide BN-PAGE was used. *Asterisks* (∗ and ∗∗) indicate subcomplexes of Tim22 and Tim23. The *box* indicates the nonspecific band.

We examined the importance of conserved cysteine residues 213 and 222 of the TLF domain by constructing individual changes to serine (designated C213S and C222S, Fig. S3). Aim32 and mutant proteins were overexpressed ∼10-fold from a 2-micron plasmid (Fig. 8*B*) because expression from a centromeric plasmid was not detected. A C-terminal FLAG tag as in Figure 6*A* was also included. These strains grew at 30^o^C but arrested growth at 37^o^C on ethanol-glycerol media (YPEG and SEG-URA), similar to the *Δaim32* strain expressing the Vec (Fig. 8*A*). Also, growth of the cysteine mutants was compromised in media supplemented with HU (Fig. 8*A* and (24)). Additional cysteine mutants were tested (C38S, C40S, and C291S), and results indicated these changes did not alter cell growth (data not shown, and (24)).

The steady-state level of mitochondrial proteins in mitochondria from the cysteine mutants was examined (Fig. 8*B*). The Aim32-FLAG proteins migrated slightly higher than Aim32 and were overexpressed ∼10-fold. Tim23, Tim22, Tim13, and Yme1 were present at increased abundance in the cysteine mutant strains (Fig. 8*B*). In contrast, the amount of Tim13, Tim22, and Tim23 was decreased in the Δ*aim32* strain (Fig. 5*A*). The abundance of other proteins in the cysteine mutants, however, was not strongly changed (Fig. 8*B*). We also tested assembly of the import complexes (Fig. 8*C*) and showed that, in the single cysteine mutants, the TIM22 and TIM23 complexes seem to have several assembly states and Yme1 complex assembly was not impaired.

**Figure 9:**
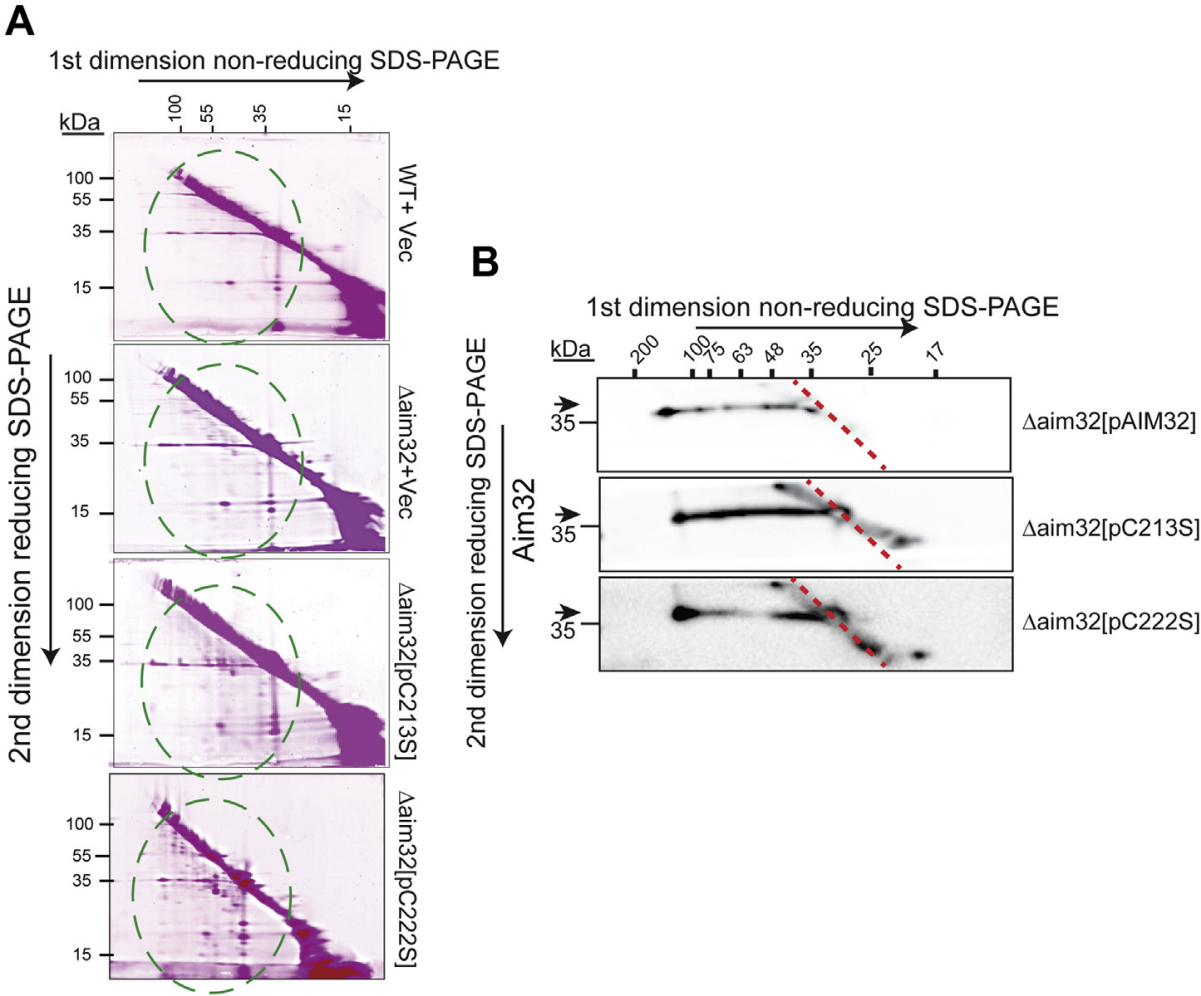
**Cysteine residues 213 and 222 of Aim32 are critical for native disulfide formation in target proteins.***A*, mitochondrial lysates were analyzed as in Figure 8*B*. The *Δaim32* strain was transformed with WT *AIM32* (pAIM32) or *aim32* mutants with single cysteine to serine changes (pC213S or pC222S). *Green dashed circles* indicate regions of increased rhodamine labeling in the Aim32 cysteine mutants. *B*, as in *panel A*, except that 2DGE was performed on the indicated mitochondrial extracts and analyzed by Western blotting against Aim32. *Dashed red line* indicates the position of the diagonal. *Horizontal arrows* at the left of the panel align with the location of the signal expected for each of the individual proteins tested. Aim32, altered inheritance of mitochondria 32; 2D-DGE, two-dimensional diagonal gel electrophoresis.

Finally, we examined the mitochondrial redox status of protein thiols as in Figure 7 in the strains with the single cysteine mutants C213S and C222S. An increase in proteins with non-native disulfide was observed for the single cysteine mutants in contrast to WT and the strain that lacks Aim32 (Fig. 9*A*, green dashed circles). With 2D-DGE followed by immunoblotting against Aim32, an overabundance of disulfide-linked Aim32 heterodimers in the single cysteine mutants (Fig. 9*B*) was observed. The overexpression strain (Δ*aim32* [pAIM32-FLAG]) was included as a control strain because the expression of Aim32 is comparable across strains (Fig. 8*B*). Because we have previously shown that Aim32 interacts with multiple proteins via disulfide-linked heterodimers (Fig. 7*B*), the single cysteine mutants may be trapped via intermolecular thiol bonds with many proteins. In sum, cysteine residues, C213 and C222, of the TLF domain are important in redox modulation.

## Discussion

### Aim32 is dually localized to the mitochondrial IMS and matrix

In this study, we report the physiologic characterization of Aim32, a mitochondrial Fe-S protein that has a highly conserved C-terminal TLF domain (Fig. S3). Stegmaier *et al*. (24) recently completed a chemical characterization of Aim32 and showed that it is a bis-histidinyl coordinated [2Fe-2S] protein. They also proposed a role for Aim32 in degradation of allelochemicals. Aim32 was also localized to the mitochondrial matrix and/or IM in several studies, including large-scale screens (24, 36, 50). However, mitochondrial-specific functions for Aim32 have not been assigned.

Our analysis reveals that Aim32 is dual-localized to both the IMS and matrix, which is atypical for mitochondrial proteins. As this was unexpected, we used several complementary approaches with endogenous and tagged Aim32 proteins to confirm this localization to both mitochondrial compartments. Aim32 was identified as an Erv1-interacting protein, suggesting a role for Aim32 in the IMS. Localization of Aim32 to the IMS was corroborated by osmotic gradient experiments with intact mitochondria and with *in vitro* imported Aim32 into mitochondria. Studies with the *in vivo* site-specific (TEV protease) cleavage assay, however, confirmed localization in both the IMS and matrix.

We demonstrate that Aim32 relies mostly on the TIM23 pathway for import into the mitochondria. Aim32 contains an N-terminal mitochondrial-targeting sequence that is efficiently cleaved by MPP, in agreement with an MPP cut site predicted by MitoFates (at the 13th amino acid). Aim32 import also requires a Δψ to initiate translocation across the TIM23 translocon. Thus, MPP cleaves the mitochondrial-targeting sequence of Aim32 as it enters the matrix. An involvement of the MIA pathway for localization to the IMS cannot be excluded based on our localization studies, and reduced import in the presence of MitoBloCK-6, an Erv1-specific inhibitor (Fig. 2*H*). Only one processed form of Aim32 is detected in all our studies. Thus, the Aim32 pool is likely processed by MPP. A pathway for Aim32 import and maturation may be proposed in which a cohort of Aim32 molecules are fully imported into the matrix, processed by MPP, and assembled. Then, some processed Aim32 may halt translocation and fall back into the IMS. This “stop-transfer” of Aim32 into the IMS may be akin to MPP-processed fumarase that accumulates in the cytosol (51), with the exception that the C terminus of Aim32 completely enters the IMS. Further support of protein dual localization is exemplified by aconitase, which demonstrates highly unequal distribution of a single translation product between the cytosol and the mitochondria (52). Similar to aconitase, we hypothesize that IMS-localized Aim32 constitutes a smaller fraction of the protein pool than that found in the matrix. The possibility that the IMS-localized Aim32 species is an off-pathway/failed intermediate cannot be completely excluded. But, when Aim32 is exclusively targeted to the matrix, cells still demonstrated growth deficiency on HU-supplemented plates (data not shown), suggesting that lack of Aim32 in the IMS has functional consequences. The exact function of Aim32 in the IMS, however, remains to be determined.

The IMS-cohort of Aim32 may be regulated by the activity of molecular chaperones, and/or binding to IMS proteins (e.g., Erv1, Mia40). Aim32 localization in the IMS may be biased under specific conditions of stress. For example, human CCS1 and SOD1 import into the IMS are linked to mitochondrial ROS levels (53), and p53 localizes to mitochondria via Mia40 under stress conditions (54). Future studies to establish the relevance of Aim32 activity in both mitochondrial compartments are warranted.

### Aim32 is required for maintaining the global redox status

Although *AIM32* is not essential for viability, the protein is required for respiration at elevated temperature and cells that lack *AIM32* are petite negative, an inability to grow after mtDNA loss because of EtBr treatment (55). This is not unusual as it has been previously reported that the null mutant shows an increased propensity to lose its mitochondrial genome (56). Such dependence upon mtDNA was also seen in strains lacking key mitochondrial protein import components (57). In cells lacking *Aim32*, the change in efficiency of important import components (Fig. 4) along with potential deficiency in electron transport likely exacerbates its growth phenotype on EtBr-supplemented plates. Finally, Aim32 is absolutely required when cells are challenged with HU.

A recent genome-wide screen suggested that Aim32 was a crucial factor for the intracellular sorting of the mitochondrial IM protein Oxa1 (58); however, the mechanism is not clear. In our analyses, cells depleted for *AIM32* showed reduced steady-state levels of Oxa1, and Oxa1 complex assembly (Fig. 5). The requirement of Aim32 was not restricted to Oxa1 because steady-state levels and import of newly synthesized MIA pathway precursors were severely impacted as well (Figs. 4 and 5). Furthermore, the assembly profile of several essential protein import complexes (TIM22, TIM23^SORT^, and small TIM) was altered in the absence of functional *AIM32*. Our data strongly support a role for Aim32 in stabilization of various protein complexes, including some key import complexes such as TIM23 and TIM22. Because no effect was observed on the TOM or the SAM import complexes, it is likely that crucial component proteins of the TIM23 pathway such as Tim23, Tim17, and Tim50 serve as “clients” of Aim32. In addition, the redox status of Erv1 that is a vital component of the MIA import pathway was altered in absence of Aim32 (Fig. 6*C*). Future functional analysis, however, is needed to conclusively delineate between the two possibilities. Given that Aim32 has numerous binding partners, Aim32 may have a global role in quality control and/or stabilization of mitochondrial proteins, including the import complexes.

Our study supports a global role for Aim32 in maintaining the redox status of the mitochondrial proteome. In the absence of *AIM32*, we find that the mitochondrial thiol proteome contains an increase in aberrant thiol linkages (Fig. 7*C*). The 2D-DGE/Western blot analysis highlighted several mixed disulfide linkages formed between Aim32 and target proteins (Fig. 7*B*), and the redox status of a subset of candidate proteins investigated (i.e., Tim13, Tim50, and Osm1) was altered. Specifically, Aim32 redox-active cysteine mutants displayed an increase of the Tim22 dimer and decrease of the mature TIM22 complex (Fig. 8*C*), suggesting that the Tim22 dimer was not assembling efficiently or the TIM22 complex was destabilized (59). Similar destabilization of the TIM23^SORT^ complex was observed in the cysteine mutants. Analysis of the mitochondrial thiol proteome in the single cysteine mutants of Aim32 (C213S, C222S) revealed an increase in aberrant thiol interactions (Fig. 9*A*), along with several “trapped” mixed disulfide intermediates formed between Aim32 and target proteins (Fig. 9*B*).

Thioredoxins are known to bind to proteins, regulating their activity. For instance, thioredoxin-1 acts as a redox sensor of ASK1 and regulates its transcriptional activity (60). It is interesting that Aim32 forms stable complexes with a subset of mitochondrial proteins. This potentially could be to regulate protein activity and/or protect thiol proteins from oxidation, as is proposed for glutathionylation (61). In the absence of *AIM32* or in the Aim32 cysteine mutants, yeast are susceptible to conditions of stress (Figs. 3*D* and 8*A*). We demonstrate that a portion of matrix-localized Aim32 is physically bound to Sod2 (Fig. 6). Moreover, overexpression of *AIM32* protected yeast cells against PQ-induced oxidative damage in the absence of *SOD2*, the cells primary defense mechanism against ROS (23). Presumably, Aim32 can repair oxidized cysteine residues, and protein activity can be restored after exposure to cellular stress. Another, albeit remote, possibility is that Aim32 exhibits protein disulfide isomerase activity (akin to DsbA), helping in maintenance of proteins in their native folded state. Because the Aim32 single cysteine mutants form mixed disulfide intermediates, future studies can be designed to identify substrates and determine the molecular basis of the interaction with substrates. In sum, this study begins to dissect the role of Aim32, a TLF protein that normally resides within the mitochondrial matrix and functions in quality control.

## Experimental procedures

### Strains and site-directed mutagenesis

For pull-down analysis and MS studies, the designated yeast strains Erv1-HISPC and erv1-12-HISPC were generated. The HISPC tag consists of a protein-C epitope tag separated from a His10 tag by a 5-amino acid linker. Either *ERV1* or *erv1-12* alleles with 300 nucleotides of the 5′ promoter region were cloned into plasmid pRS425 (2 micron, *LEU2*). The HISPC tag (amino acid sequence MEDQVDPRLIDGK–GGAGG–HHHHHHHHHH; PC epitope tag underlined) was integrated at the C terminus of *AIM32* with PCR overlap extension (62). The plasmids were transformed into strain yEPL (*his3 leu2 ura3 trp1 ade8 erv1::HIS3* [pERV1:URA3 2μ]) and then the WT *ERV1* plasmid was removed by plasmid shuffling.

*AIM32* was PCR-amplified from yeast genomic DNA isolated from the WT (GA74-1A) strain and the 3′ primer introduced a 3XFLAG tag to the C terminus. The fragment was cloned into vector pRS316 that contains the *GPD1* promoter and *PGK1* terminator, designated [pAIM32], and transformed into Δ*aim32* yeast strain (Fig. S4*D*). Mutagenesis was carried out according to Agilent's site-directed mutagenesis protocols. Mutagenized constructs [pC213S], [pC222S], [pC213, 222S], [pH249, 253A], and [pW278STOP] were generated in the aforementioned pRS316 vector with FLAG-tagged Aim32 and transformed into the Δ*aim32* yeast strain.

The parental *S. cerevisiae* yeast strain used in the study was GA74-1A (Table S1). The *Δaim32::HIS* strain was generated by deletion of *AIM32* with *HIS3MX* in GA74-1A using standard procedures (63). Standard yeast genetics were used to generate the strains and for synthetic lethal analysis (64).

### Subcellular fractionation and mitochondrial assays

Subcellular fractionation from spheroplasts was performed as previously described (65). The fractions were separated by differential centrifugation, and an equal amount of each fraction was separated by SDS-PAGE followed by immunoblot analysis.

Mitochondria were purified from yeast cells grown in media with glucose or ethanol/glycerol (66). Proteins for import studies were synthesized by the TNT Quick Coupled Transcription/Translation kits (Promega) in the presence of [^35^S] methionine. The radiolabeled precursor was incubated with isolated mitochondria and an import time course was performed. Where indicated, the ΔΨ was dissipated with 5 mM carbonyl cyanide m-chlorophenyl hydrazone (CCCP). Nonimported radiolabeled protein was removed by treatment with 100 μg/ml trypsin for 15 min on ice, and trypsin was inhibited with 200 μg/ml of soybean trypsin inhibitor for 30 min on ice. Samples were separated by SDS-PAGE and visualized using autoradiography. Mitochondria were purified from the Erv1-His_6_ strain, and pull-down experiments were performed as previously described (10).

Mitochondrial fractionation was performed as previously described (65). For intact mitochondria, 300 μg of isolated mitochondria were incubated in 0.6 M sorbitol and 20 mM Hepes-KOH, pH 7.4. To generate mitoplasts, 300 μg of isolated mitochondria were incubated in 0.03 M sorbitol and 20 mM Hepes-KOH, pH 7.4. As indicated, 100 μg/ml of Proteinase K and 0.1% Triton X-100 were added. Samples were centrifuged at 14,000*g* for 10 min to separate the pellet and supernatant. The supernatants were precipitated with 20% trichloroacetic acid and resuspended in SDS sample buffer.

Carbonate extraction was performed as described previously (65). Briefly, 200 μg of mitochondria was incubated in 200 μl of 0.1 M Na_2_CO_3_ at the indicated pH for 15 min on ice. Samples were centrifuged at 14,000*g* for 10 min to separate the pellet and supernatant. The supernatants were precipitated with 20% (wt/vol) TCA, and the pellet and supernatant fractions were resuspended in equal volumes of SDS-sample buffer.

One-dimensional BN-PAGE was performed as described previously (12). For 2D Blue Native/SDS-PAGE (65) before resolution by second dimension by SDS-PAGE, the BN-PAGE gel was soaked in 1% (wt/vol) SDS, 1% (vol/vol) β-mercaptoethanol for 30 min at 50 °C, and individual lanes isolated with a razor blade, embedded in a 4% stacking gel.

For indirect thiol trapping assay, isolated mitochondria from the respective strains were treated with 150 mM mmPEG (22713 Thermo Fisher) for 1 h at room temperature (RT) in the dark. In control reactions, mitochondria were treated with either 20 mM DTT for 15 min at 65 °C or 50 mM IAA for 10 min at 30 °C. Samples were analyzed by nonreducing SDS-PAGE.

### TEV protease cleavage assay

Plasmids on the pRS416 backbone containing a matrix-localized Su9-TEV protease and IMS-localized CYB2[1-220]-TEV protease under the control of the GAL1 promoter were obtained for protease expression (37). The centromeric pRS315 vector was used for plasmid expression of Nuc1 or Aim32 proteins. The *Δaim32* strains expressing Aim32-3XFLAG-TEV-3HA, Sod2-3XFLAG-TEV-3HA, and WT strain endogenously tagged with Nuc1-3XFLAG-TEV-3HA were transformed with plasmids expressing matrix-localized Su9-TEV or IMS-localized CYB2[1-220]-TEV. Yeast strains were grown in the appropriate growth media and harvested in the midlog phase. Whole-cell extracts were analyzed by immunoblotting with FLAG, HA, Tim44, Hsp70, and Aim32 antibodies.

### 2D-DGE

Two-dimensional DGE was performed as described previously (67). Briefly, mitochondria (5 mg/ml) were solubilized with 20 mM Hepes-KOH, pH 7.4, 10% glycerol, 50 mM NaCl, 1 mM EDTA, and 2.5 mM MgCl_2_ supplemented with 1% (w/v) digitonin and protease inhibitors. Lysates were treated with 500 nM IAA-rhodamine at RT followed by resolution in the first dimension on a nonreducing SDS gel. Individual lanes isolated with a razor blade were soaked in 1% (wt/vol) SDS and 1% (vol/vol) β-mercaptoethanol for 30 min at 50 °C, embedded in a 4% stacking gel, and resolved in the second dimension by SDS-PAGE. Gels were visualized using the Sapphire Biomolecular Imager (Azure Biosystems).

### Protein–protein binding assays and MS

Pull-down experiments designed to detect possible complex formation between Erv1 and Aim32 or Osm1-utilizing His tags were described previously (12). Briefly, mitochondria were purified, solubilized in 1% digitonin, and subjected to pull-down experiments with Ni^2+^ agarose. FLAG immunoprecipitations were performed using the ANTI-FLAG M2 affinity gel (Sigma Aldrich) using the manufacturers' guidelines.

CNAP pull-downs were performed as described in Claypool *et al.* (32). Briefly, detergent solubilization of mitochondria (5 mg/ml) was performed with 20 mM Hepes-KOH, pH 7.4, 20 mM imidazole, 10% glycerol, 100 mM NaCl, and 1 mM CaCl_2_, supplemented with 1.5% (wt/vol) digitonin (Biosynth International, Inc.) and protease inhibitors (1 mM PMSF, 10 μM leupeptin, 2 μM pepstatin A, and 10 μM chymostatin). Insoluble material was removed at 20,000*g* for 30 min at 4 °C and extracts transferred to tubes containing 8-ml lysis buffer base with added protease inhibitors and 0.8 ml Ni-NTA resin (QIAGEN). After rotating at 4 °C for 1 h, the contents were poured into a column, and the flow through collected. After two washes with 8-ml lysis buffer base containing 0.1% (wt/vol) digitonin, bound material was directly eluted into separate columns containing 1 ml equilibrated anti-PC resin (Roche; equilibration buffer 20 mM Hepes-KOH, pH 7.4, 100 mM NaCl, and 1 mM CaCl_2_) with 8 ml Ni2+ elution buffer (20 mM Hepes-KOH, pH 7.4, 300 mM imidazole, 10% glycerol, 100 mM NaCl, 1 mM CaCl_2_, and 0.1% digitonin). After 1-h rotating at 4 °C, the flow through was collected and the columns washed twice with 10-ml anti-PC wash buffer (anti-PC equilibration buffer with 0.1% digitonin). To elute bound material, the columns were capped and 1 ml of EDTA elution solution (20 mM Hepes-KOH, pH 7.4, 100 mM NaCl, 5 mM EDTA, and 0.1% digitonin) was added to the anti-PC resin. Four elutions were performed. The first elution consisted of 15 min at 4 °C and then 15 min at RT; the remaining three elutions were at RT. Eluates were directly pooled in Amicon filtration devices, 10,000 molecular weight cut-off kept on ice, and the pooled eluates concentrated following the provided guidelines (Millipore). Concentrated CNAPed product derived from 4 and 10 mg starting material was analyzed in the preparative 1D-PAGE analyses, and proteins revealed with the SYPRO Ruby stain and protocol (Invitrogen). Gels were imaged and analyzed with PDQuest (Bio-Rad Laboratories) software. Protein bands of interest were excited by a spot-excision robot (ProteomeWorks; Bio-Rad Laboratories) and deposited into 96-well plates. Gel bands were digested with sequencing-grade trypsin (Promega), and the resulting tryptic peptides were extracted using the standard protocol (68). LC–MS/MS of peptide mixtures was performed on a QSTAR Pulsar XL (QqTOF) mass spectrometer (Applied Biosystems) equipped with nanoelectrospray interface (Protana) and LC Packings nano-LC system. All searches were performed against the Swiss-Prot protein sequence database.

### LC-MS analysis

The protein mixtures were reduced, alkylated, and digested by the sequential addition of trypsin and lys-C proteases. Afterward, samples were desalted using Pierce C18 tips, eluted in 40% acetonitrile, and dried and resuspended in 5% formic acid. Desalted samples were separated on C18 reversed phase (1.9 μM, 100A pores, Dr Maisch GmbH) columns packed with 25 cm of resin in a 75-μM inner diameter fused silica capillary, as described elsewhere (69). Digested peptides were fractionated online using a 140-min water–acetonitrile gradient with 3% dimethyl sulfoxide ionized using electrospray ionization by application of a distal 2.2 kV. Ionized peptides were interrogated via MS/MS in a Thermo Orbitrap Fusion Lumos. For discovery acquisitions, data-dependent acquisition was utilized with an MS1 scan resolution of 120,000 and MS2 resolution of 15,000 and a cycle time of 3 s. Data analysis was performed using the Integrated Proteomics pipeline 2 (Integrated Proteomics Applications). MS/MS spectra were searched using the ProLuCID algorithm and peptide-to-spectrum matches were organized and filtered based on decoy database-estimated false discovery rate of <1% using the DTASelect algorithm. Database searching was performed using a *S. cerevisiae* yeast database downloaded from *Saccharomyces* Genome Database on 2-19-2016 (12,080 entries). Specificity of Trypsin and lysc with cleaving C-terminal to arginine or lysine residues was accepted, while one missed cleavage was allowed. Carbamidomethylation (C2H3NO, 57.02146 Da) was the fixed modifications, while no variable modification in these data was considered. Mass tolerance for both the precursor and fragment ions was set to 15 ppm

### Sequence alignments

Sequence alignments were generated using the CLC Workbench software (QIAGEN). Calculation of the information content in sequence logos is provided in the original article (70). Multiple protein sequence alignments were generated using PROMALS, a method that improves alignment quality by using additional homologs from PSI-BLAST searches and secondary structure predictions from PSIPRED (71).

### Assessment of mitochondrial Δψ

The ΔΨ of isolated yeast mitochondria was assessed by measuring the fluorescence quenching of the potential-sensitive dye DiSC_3_ (5) (Molecular Probes) as described previously (72). The measurements were performed using a FlexStation plate reader (Molecular Devices) controlled with the SoftMax Pro software package (Molecular Devices) with excitation at 622 nm and emission at 670 nm at 25 °C. Mitochondria (21 μg/ml) in the buffer (0.6 M sorbitol, 1% BSA, 10 mM MgCl_2_, 0.5 mM EDTA, 20 mM KPO_4_, pH 7.4, and 2 mM NADH) was added to the cuvette, followed by DiSC_3_ (5) (final concentration of 167 nM in ethanol) addition, and the fluorescence was measured. Mitochondria were subsequently uncoupled with CCCP (final concentration of 20 μM in ethanol). The difference in the fluorescence before and after the addition of CCCP represents a relative measurement of ΔΨ.

### Antibodies

Most of the antibodies used in this work were generated by the vendor Pacific Immunology from recombinant proteins in the Koehler laboratory and have been described previously. Other antibodies used were as follows: anti-Cox-4, anti-Rip1-FeS, anti-Qcr10, and anti-Oxa1 (a gift of Dr Rosemary Stuart, Marquette University), and horseradish peroxidase–conjugated secondary antibodies (Thermo Fisher Scientific).

### Statistical analyses

Data were plotted using GraphPad Prism, version 5.02. Statistical significance of observations was determined using unpaired t tests (two tailed), unless otherwise noted.

## Data availability

All data are contained within the article. The mass spectrometry proteomics data (AimFLAG IP) are available via ProteomeXchange (73) with identifier PXD021662 and the *erv1-12*HISPC pull-down data via accession number JPST001221 (submitted to jPOST (74)).

## Supporting information

This article contains supporting information (10, 12, 75, 76, 77).

## Conflict of interest

The authors declare that they have no conflicts of interest with the contents of this article.

## References

[1] Chacinska A., Pfannschmidt S., Wiedemann N., Kozjak V., Sanjuan Szklarz L.K., Schulze-Specking A., Truscott K.N., Guiard B., Meisinger C., Pfanner N. (2004). Essential role of Mia40 in import and assembly of mitochondrial intermembrane space proteins. EMBO J..

[2] Allen S., Balabanidou V., Sideris D.P., Lisowsky T., Tokatlidis K. (2005). Erv1 mediates the Mia40-dependent protein import pathway and provides a functional link to the respiratory chain by shuttling electrons to cytochrome c. J. Mol. Biol..

[3] Mesecke N., Terziyska N., Kozany C., Baumann F., Neupert W., Hell K., Herrmann J.M. (2005). A disulfide relay system in the intermembrane space of mitochondria that mediates protein import. Cell.

[4] Tienson H.L., Dabir D.V., Neal S.E., Loo R., Hasson S.A., Boontheung P., Kim S.K., Loo J.A., Koehler C.M. (2009). Reconstitution of the mia40-erv1 oxidative folding pathway for the small tim proteins. Mol. Biol. Cell.

[5] Koehler C.M., Tienson H.L. (2009). Redox regulation of protein folding in the mitochondrial intermembrane space. Biochim. Biophys. Acta.

[6] Chacinska A., Koehler C.M., Milenkovic D., Lithgow T., Pfanner N. (2009). Importing mitochondrial proteins: Machineries and mechanisms. Cell.

[7] Sideris D.P., Tokatlidis K. (2010). Oxidative protein folding in the mitochondrial intermembrane space. Antioxid. Redox Signal.

[8] Weckbecker D., Longen S., Riemer J., Herrmann J.M. (2012). Atp23 biogenesis reveals a chaperone-like folding activity of Mia40 in the IMS of mitochondria. EMBO J..

[9] Bien M., Longen S., Wagener N., Chwalla I., Herrmann J.M., Riemer J. (2010). Mitochondrial disulfide bond formation is driven by intersubunit electron transfer in Erv1 and proofread by glutathione. Mol. Cell.

[10] Dabir D.V., Leverich E.P., Kim S.K., Tsai F.D., Hirasawa M., Knaff D.B., Koehler C.M. (2007). A role for cytochrome c and cytochrome c peroxidase in electron shuttling from Erv1. EMBO J..

[11] Bihlmaier K., Mesecke N., Terziyska N., Bien M., Hell K., Herrmann J.M. (2007). The disulfide relay system of mitochondria is connected to the respiratory chain. J. Cell Biol..

[12] Neal S.E., Dabir D.V., Wijaya J., Boon C., Koehler C.M. (2017). Osm1 facilitates the transfer of electrons from Erv1 to fumarate in the redox-regulated import pathway in the mitochondrial intermembrane space. Mol. Biol. Cell.

[13] Fraga H., Bech-Serra J.J., Canals F., Ortega G., Millet O., Ventura S. (2014). The mitochondrial intermembrane space oxireductase Mia40 funnels the oxidative folding pathway of the cytochrome c oxidase assembly protein Cox19. J. Biol. Chem..

[14] Hudson D.A., Thorpe C. (2015). Mia40 is a facile oxidant of unfolded reduced proteins but shows minimal isomerase activity. Arch. Biochem. Biophys..

[15] Fischer M., Horn S., Belkacemi A., Kojer K., Petrungaro C., Habich M., Ali M., Kuttner V., Bien M., Kauff F., Dengjel J., Herrmann J.M., Riemer J. (2013). Protein import and oxidative folding in the mitochondrial intermembrane space of intact mammalian cells. Mol. Biol. Cell.

[16] Neal S.E., Dabir D.V., Tienson H.L., Horn D.M., Glaeser K., Ogozalek Loo R.R., Barrientos A., Koehler C.M. (2015). Mia40 protein serves as an electron sink in the mia40-erv1 import pathway. J. Biol. Chem..

[17] Hu J., Dong L., Outten C.E. (2008). The redox environment in the mitochondrial intermembrane space is maintained separately from the cytosol and matrix. J. Biol. Chem..

[18] Ayer A., Tan S.X., Grant C.M., Meyer A.J., Dawes I.W., Perrone G.G. (2010). The critical role of glutathione in maintenance of the mitochondrial genome. Free Radic. Biol. Med..

[19] Kojer K., Bien M., Gangel H., Morgan B., Dick T.P., Riemer J. (2012). Glutathione redox potential in the mitochondrial intermembrane space is linked to the cytosol and impacts the Mia40 redox state. EMBO J..

[20] Ayer A., Fellermeier S., Fife C., Li S.S., Smits G., Meyer A.J., Dawes I.W., Perrone G.G. (2012). A genome-wide screen in yeast identifies specific oxidative stress genes required for the maintenance of sub-cellular redox homeostasis. PLoS One.

[21] Palma F.R., He C., Danes J.M., Paviani V., Coelho D.R., Gantner B.N., Bonini M.G. (2020). Mitochondrial superoxide dismutase: What the established, the intriguing, and the novel reveal about a key cellular redox switch. Antioxid. Redox Signal.

[22] Tu B.P., Weissman J.S. (2004). Oxidative protein folding in eukaryotes: Mechanisms and consequences. J. Cell Biol..

[23] Laleve A., Vallieres C., Golinelli-Cohen M.P., Bouton C., Song Z., Pawlik G., Tindall S.M., Avery S.V., Clain J., Meunier B. (2016). The antimalarial drug primaquine targets Fe-S cluster proteins and yeast respiratory growth. Redox Biol..

[24] Stegmaier K., Blinn C.M., Bechtel D.F., Greth C., Auerbach H., Muller C.S., Jakob V., Reijerse E.J., Netz D.J.A., Schunemann V., Pierik A.J. (2019). Apd1 and Aim32 are prototypes of bis-histidinyl-coordinated non-Rieske [2Fe-2S] proteins. J. Am. Chem. Soc..

[25] Lu J., Holmgren A. (2014). The thioredoxin superfamily in oxidative protein folding. Antioxid. Redox Signal.

[26] Hanschmann E.M., Godoy J.R., Berndt C., Hudemann C., Lillig C.H. (2013). Thioredoxins, glutaredoxins, and peroxiredoxins--molecular mechanisms and health significance: From cofactors to antioxidants to redox signaling. Antioxid. Redox Signal.

[27] Groitl B., Jakob U. (2014). Thiol-based redox switches. Biochim. Biophys. Acta.

[28] Couturier J., Przybyla-Toscano J., Roret T., Didierjean C., Rouhier N. (2015). The roles of glutaredoxins ligating Fe-S clusters: Sensing, transfer or repair functions?. Biochim. Biophys. Acta.

[29] Huber-Wunderlich M., Glockshuber R. (1998). A single dipeptide sequence modulates the redox properties of a whole enzyme family. Fold Des..

[30] Maskos K., Huber-Wunderlich M., Glockshuber R. (2003). DsbA and DsbC-catalyzed oxidative folding of proteins with complex disulfide bridge patterns *in vitro* and *in vivo*. J. Mol. Biol..

[31] Mossner E., Huber-Wunderlich M., Glockshuber R. (1998). Characterization of Escherichia coli thioredoxin variants mimicking the active-sites of other thiol/disulfide oxidoreductases. Protein Sci..

[32] Claypool S.M., Oktay Y., Boontheung P., Loo J.A., Koehler C.M. (2008). Cardiolipin defines the interactome of the major ADP/ATP carrier protein of the mitochondrial inner membrane. J. Cell Biol..

[33] Rainey R.N., Glavin J.D., Chen H.W., French S.W., Teitell M.A., Koehler C.M. (2006). A new function in translocation for the mitochondrial i-AAA protease Yme1: Import of polynucleotide phosphorylase into the intermembrane space. Mol. Cell Biol..

[34] Fukasawa Y., Tsuji J., Fu S.C., Tomii K., Horton P., Imai K. (2015). MitoFates: Improved prediction of mitochondrial targeting sequences and their cleavage sites. Mol. Cell Proteomics.

[35] Deng K., Zhang L., Kachurin A.M., Yu L., Xia D., Kim H., Deisenhofer J., Yu C.A. (1998). Activation of a matrix processing peptidase from the crystalline cytochrome bc1 complex of bovine heart mitochondria. J. Biol. Chem..

[36] Vogtle F.N., Burkhart J.M., Gonczarowska-Jorge H., Kucukkose C., Taskin A.A., Kopczynski D., Ahrends R., Mossmann D., Sickmann A., Zahedi R.P., Meisinger C. (2017). Landscape of submitochondrial protein distribution. Nat. Commun..

[37] Kondo-Okamoto N., Shaw J.M., Okamoto K. (2003). Mmm1p spans both the outer and inner mitochondrial membranes and contains distinct domains for targeting and foci formation. J. Biol. Chem..

[38] Faber K.N., Kram A.M., Ehrmann M., Veenhuis M. (2001). A novel method to determine the topology of peroxisomal membrane proteins *in vivo* using the tobacco etch virus protease. J. Biol. Chem..

[39] Buttner S., Eisenberg T., Carmona-Gutierrez D., Ruli D., Knauer H., Ruckenstuhl C., Sigrist C., Wissing S., Kollroser M., Frohlich K.U., Sigrist S., Madeo F. (2007). Endonuclease G regulates budding yeast life and death. Mol. Cell.

[40] Fujiki Y., Hubbard A.L., Fowler S., Lazarow P.B. (1982). Isolation of intracellular membranes by means of sodium carbonate treatment: Application to endoplasmic reticulum. J. Cell Biol..

[41] Gevorkyan-Airapetov L., Zohary K., Popov-Celeketic D., Mapa K., Hell K., Neupert W., Azem A., Mokranjac D. (2009). Interaction of Tim23 with Tim50 Is essential for protein translocation by the mitochondrial TIM23 complex. J. Biol. Chem..

[42] Dabir D.V., Hasson S.A., Setoguchi K., Johnson M.E., Wongkongkathep P., Douglas C.J., Zimmerman J., Damoiseaux R., Teitell M.A., Koehler C.M. (2013). A small molecule inhibitor of redox-regulated protein translocation into mitochondria. Dev. Cell.

[43] Pei J., Kim B.H., Tang M., Grishin N.V. (2007). PROMALS web server for accurate multiple protein sequence alignments. Nucleic Acids Res..

[44] Slonimski P.P., Perrodin G., Croft J.H. (1968). Ethidium bromide induced mutation of yeast mitochondria: Complete transformation of cells into respiratory deficient non-chromosomal “petites”. Biochem. Biophys. Res. Commun..

[45] Tang H.M., Pan K., Kong K.Y., Hu L., Chan L.C., Siu K.L., Sun H., Wong C.M., Jin D.Y. (2015). Loss of APD1 in yeast confers hydroxyurea sensitivity suppressed by Yap1p transcription factor. Sci. Rep..

[46] Bakeeva L.E., Derevianchenko I.G., Konoshenko G.I., Mokhova E.N. (1983). [Interaction of diS-C3-(5) and ethylrhodamine with lymphocyte mitochondria]. Biokhimiia.

[47] Habich M., Salscheider S.L., Murschall L.M., Hoehne M.N., Fischer M., Schorn F., Petrungaro C., Ali M., Erdogan A.J., Abou-Eid S., Kashkar H., Dengjel J., Riemer J. (2019). Vectorial import via a metastable disulfide-linked complex allows for a quality control step and import by the mitochondrial disulfide relay. Cell Rep..

[48] Ang S.K., Zhang M., Lodi T., Lu H. (2014). Mitochondrial thiol oxidase Erv1: Both shuttle cysteine residues are required for its function with distinct roles. Biochem. J..

[49] Hill B.G., Reily C., Oh J.Y., Johnson M.S., Landar A. (2009). Methods for the determination and quantification of the reactive thiol proteome. Free Radic. Biol. Med..

[50] Morgenstern M., Stiller S.B., Lubbert P., Peikert C.D., Dannenmaier S., Drepper F., Weill U., Hoss P., Feuerstein R., Gebert M., Bohnert M., van der Laan M., Schuldiner M., Schutze C., Oeljeklaus S. (2017). Definition of a high-confidence mitochondrial proteome at quantitative scale. Cell Rep..

[51] Stein I., Peleg Y., Even-Ram S., Pines O. (1994). The single translation product of the FUM1 gene (fumarase) is processed in mitochondria before being distributed between the cytosol and mitochondria in Saccharomyces cerevisiae. Mol. Cell Biol..

[52] Regev-Rudzki N., Karniely S., Ben-Haim N.N., Pines O. (2005). Yeast aconitase in two locations and two metabolic pathways: Seeing small amounts is believing. Mol. Biol. Cell.

[53] Sturtz L.A., Diekert K., Jensen L.T., Lill R., Culotta V.C. (2001). A fraction of yeast Cu,Zn-superoxide dismutase and its metallochaperone, CCS, localize to the intermembrane space of mitochondria. A physiological role for SOD1 in guarding against mitochondrial oxidative damage. J. Biol. Chem..

[54] Zhuang J., Wang P.Y., Huang X., Chen X., Kang J.G., Hwang P.M. (2013). Mitochondrial disulfide relay mediates translocation of p53 and partitions its subcellular activity. Proc. Natl. Acad. Sci. U. S. A..

[55] Goldring E.S., Grossman L.I., Krupnick D., Cryer D.R., Marmur J. (1970). The petite mutation in yeast. Loss of mitochondrial deoxyribonucleic acid during induction of petites with ethidium bromide. J. Mol. Biol..

[56] Hess D.C., Myers C.L., Huttenhower C., Hibbs M.A., Hayes A.P., Paw J., Clore J.J., Mendoza R.M., Luis B.S., Nislow C., Giaever G., Costanzo M., Troyanskaya O.G., Caudy A.A. (2009). Computationally driven, quantitative experiments discover genes required for mitochondrial biogenesis. PLoS Genet..

[57] Dunn C.D., Jensen R.E. (2003). Suppression of a defect in mitochondrial protein import identifies cytosolic proteins required for viability of yeast cells lacking mitochondrial DNA. Genetics.

[58] Hansen K.G., Aviram N., Laborenz J., Bibi C., Meyer M., Spang A., Schuldiner M., Herrmann J.M. (2018). An ER surface retrieval pathway safeguards the import of mitochondrial membrane proteins in yeast. Science.

[59] Okamoto H., Miyagawa A., Shiota T., Tamura Y., Endo T. (2014). Intramolecular disulfide bond of Tim22 protein maintains integrity of the TIM22 complex in the mitochondrial inner membrane. J. Biol. Chem..

[60] Saitoh M., Nishitoh H., Fujii M., Takeda K., Tobiume K., Sawada Y., Kawabata M., Miyazono K., Ichijo H. (1998). Mammalian thioredoxin is a direct inhibitor of apoptosis signal-regulating kinase (ASK) 1. EMBO J..

[61] Brunati A.M., Pagano M.A., Bindoli A., Rigobello M.P. (2010). Thiol redox systems and protein kinases in hepatic stellate cell regulatory processes. Free Radic. Res..

[62] Ho S.N., Hunt H.D., Horton R.M., Pullen J.K., Pease L.R. (1989). Site-directed mutagenesis by overlap extension using the polymerase chain reaction. Gene.

[63] Gueldener U., Heinisch J., Koehler G.J., Voss D., Hegemann J.H. (2002). A second set of loxP marker cassettes for Cre-mediated multiple gene knockouts in budding yeast. Nucleic Acids Res..

[64] Guthrie C., Fink G.R. (1991). Guide to yeast genetics and molecular biology. Methods Enzymol..

[65] Claypool S.M., McCaffery J.M., Koehler C.M. (2006). Mitochondrial mislocalization and altered assembly of a cluster of Barth syndrome mutant tafazzins. J. Cell Biol..

[66] Glick B.S., Pon L.A. (1995). Isolation of highly purified mitochondria from Saccharomyces cerevisiae. Methods Enzymol..

[67] Sommer A., Traut R.R. (1974). Diagonal polyacrylamide-dodecyl sulfate gel electrophoresis for the identification of ribosomal proteins crosslinked with methyl-4-mercaptobutyrimidate. Proc. Natl. Acad. Sci. U. S. A..

[68] Shevchenko A., Wilm M., Vorm O., Jensen O.N., Podtelejnikov A.V., Neubauer G., Shevchenko A., Mortensen P., Mann M. (1996). A strategy for identifying gel-separated proteins in sequence databases by MS alone. Biochem. Soc. Trans..

[69] Jami-Alahmadi Y., Pandey V., Mayank A.K., Wohlschlegel J.A. (2021). A robust method for packing high resolution C18 RP-nano-HPLC columns. J. Vis. Exp..

[70] Schneider T.D., Stephens R.M. (1990). Sequence logos: A new way to display consensus sequences. Nucleic Acids Res..

[71] Pei J., Grishin N.V. (2007). PROMALS: Towards accurate multiple sequence alignments of distantly related proteins. Bioinformatics.

[72] Geissler A., Krimmer T., Bomer U., Guiard B., Rassow J., Pfanner N. (2000). Membrane potential-driven protein import into mitochondria. The sorting sequence of cytochrome b_2_ modulates the deltaY-dependence of translocation of the matrix-targeting sequence. Mol. Biol. Cell.

[73] Deutsch E.W., Bandeira N., Sharma V., Perez-Riverol Y., Carver J.J., Kundu D.J., Garcia-Seisdedos D., Jarnuczak A.F., Hewapathirana S., Pullman B.S., Wertz J., Sun Z., Kawano S., Okuda S., Watanabe Y. (2020). The ProteomeXchange consortium in 2020: Enabling ‘big data’ approaches in proteomics. Nucleic Acids Res..

[74] Okuda S., Watanabe Y., Moriya Y., Kawano S., Yamamoto T., Matsumoto M., Takami T., Kobayashi D., Araki N., Yoshizawa A.C., Tabata T., Sugiyama N., Goto S., Ishihama Y. (2017). jPOSTrepo: an international standard data repository for proteomes. Nucleic Acids Res..

[75] Hennig B., Koehler H., Neupert W. (1983). Receptor sites involved in posttranslational transport of apocytochrome c into mitochondria: specificity, affinity, and number of sites. Proc. Natl. Acad. Sci. U. S. A..

[76] Hwang D.K., Claypool S.M., Leuenberger D., Tienson H.L., Koehler C.M. (2007). Tim54p connects inner membrane assembly and proteolytic pathways in the mitochondrion. J. Cell Biol..

[77] Neal S.E., Dabir D.V., Wijaya J., Boon C., Koehler C.M. (2017). Osm1 facilitates the transfer of electrons from Erv1 to fumarate in the redox-regulated import pathway in the mitochondrial intermembrane space. Mol. Biol. Cell.

