# Supplementary material for: Aim32 is a dual-localized 2Fe-2S mitochondrial protein that functions in redox quality control
Source: J Biol Chem. 2021 Aug 28;297(4):101135. doi: 10.1016/j.jbc.2021.101135 (PMC8482512; doi:10.1016/j.jbc.2021.101135)
Supplement: Figures S1–S5 and Tables S1 and S2 [file mmc1.docx]

**Supplementary Information**

**Supplementary Table**

Table S1. Yeast strains used in this study

| **Strain** | **Genotype** | **Source** |
| --- | --- | --- |
| WT (GA74-1A) | MATα *his3-11,15 leu2 ura3 trp1 ade8 rho+ mit+* | (1) |
| *erv1-12* | MATα *his3-11,15 leu2 ura3 trp1 ade8 erv1::HIS3* [p*erv1-12:TRP1* CEN | (2) |
| Erv1-His | MATα *his3-11,15 leu2 ura3 trp1 ade8 erv1::HIS3* [p*ERV1-10xHis:LEU2* 2μ] | (2) |
| yEPL1 | MATα *his3-11,15 leu2 ura3 trp1 ade8 erv1::HIS3* [pERV1:URA3 2μ] | (2) |
| Erv1-HISPC | MATα *his3-11,15 leu2 ura3 trp1 ade8 erv1::HIS3* [p*Erv1- PC10xHis:LEU2* 2μ] | This study |
| *erv1-12-*HISPC | MATα *his3-11,15 leu2 ura3 trp1 ade8 erv1::HIS3* [p*erv1- 12 PC10xHis:LEU2* 2μ] | This study |
| *Δtim54* | MATα *his3-11,15 leu2 ura3 trp1 ade8 tim54::HIS3* | (3) |
| *tim23-2* | MATα *his3-11,15 leu2 ura3 trp1 ade8 tim23-2: TRP1* | (4) |
| *Δosm1* | MATα *his3-11,15 leu2 ura3 trp1 ade8 osm1::LEU2* | (5) |
| Osm1-His | MATα *his3-11,15 leu2 ura3 trp1 ade8 osm1::LEU2* [p*OSM1- 10xHis:TRP1* 2μ] | This study |
| *Δaim32* | MATα *his3-11,15 leu2 ura3 trp1 ade8 aim32::HIS3* | This study |
| *Δaim32* [pAIM32] | MATα *his3-11,15 leu2 ura3 trp1 ade8 aim32::HIS3*  [*pRS316GPD-AIM32 3XFLAG-PGK*] | This study |
| *Δaim32* [pC213, 222S] | MATα *his3-11,15 leu2 ura3 trp1 ade8 aim32::HIS3* [*pRS316GPD-AIM32 C213, 222S 3XFLAG-PGK*] | This study |
| *Δaim32* [pH249, 253A] | MATα *his3-11,15 leu2 ura3 trp1 ade8 aim32::HIS3* [*pRS316GPD-AIM32 H249, 253A 3XFLAG-PGK*] | This study |
| *Δaim32* [pWSTOP] | MATα *his3-11,15 leu2 ura3 trp1 ade8 aim32::HIS3* [*pRS316GPD-AIM32 WSTOP 3XFLAG-PGK*] | This study |
| *Δaim32* [pC213S] | MATα *his3-11,15 leu2 ura3 trp1 ade8 aim32::HIS3* [*pRS316GPD-AIM32 C213S 3XFLAG-PGK*] | This study |
| *Δaim32* [pC222S] | MATα *his3-11,15 leu2 ura3 trp1 ade8 aim32::HIS3* [*pRS316GPD-AIM32 C222S 3XFLAG-PGK*] | This study |
| Aim32-HisPC | MATα *his3-11,15 leu2 ura3 trp1 ade8* [p*AIM32- PC10xHis:LEU2* 2μ] | This study |
| Tim23-His | MATα *his3 leu2 ade8 trp1 ura3 TIM23-HIS10:HISMX6* | This study |
| Tim17-His | MATα *his3 leu2 ade8 trp1 ura3 TIM17-HIS10:HISMX6* | This study |
| Nuc1-3XFLAG-TEV-3XHA | MATa *his3-11,15 leu2 ura3 trp1 ade8, Nuc1-3XFLAG-TEV-3XHA::KanMX6* | This study |
| Nuc1-3XFLAG-TEV-3XHA, pCYB2[1-220]-TEV Protease | MATa *his3-11,15 leu2 ura3 trp1 ade8 Nuc1-3XFLAG-TEV-3XHA::KanMX6* [*pRS416GAL1 CYB2[1-220]-TEV Protease: URA3*] | This study |
| Nuc1-3XFLAG-TEV-3XHA, pSu9-TEV Protease | MATa *his3-11,15 leu2 ura3 trp1 ade8 Nuc1-3XFLAG-TEV-3XHA::KanMX6* [*pRS416 GAL1 Su9-TEV Protease:URA3*] | This study |
| *Δaim32*[pAim32-3XFLAG-TEV-3XHA] | MATα *his3-11,15 leu2 ura3 trp1 ade8 aim32::HIS3* [p*RS315 Aim32-3XFLAG-TEV-3XHA: LEU2*] | This study |
| *Δaim32* [pAim32-3XFLAG-TEV-3XHA], pCYB2[1-220]-TEV Protease | MATα *his3-11,15 leu2 ura3 trp1 ade8 aim32::HIS3*  [*pRS315 Aim32-3XFLAG-TEV-3XHA:LEU2*] [p*RS416 GAL1 CYB2[1-220]-TEV Protease: URA3*] | This study |
| *Δaim32* [pAim32-3XFLAG-TEV-3XHA], pSu9-TEV Protease | MATα *his3-11,15 leu2 ura3 trp1 ade8 aim32::HIS3*  [p*RS315 Aim32-3XFLAG-TEV-3XHA: LEU2*] [p*RS416 GAL1Su9-TEV Protease: URA3*] | This study |
| GA74-1A [Sod2 FLAG-TEV-HA], pSu9-TEV Protease | MATα *his3-11,15 leu2 ura3 trp1 ade8 rho+ mit+* [p*RS425 Sod2-3XFLAG-TEV-3XHA: LEU2*] [p*RS416 GAL1Su9-TEV Protease: URA3*] | This study |
| GA74-1A [Sod2 FLAG-TEV-HA], pCYB2[1-220]-TEV Protease | MATα *his3-11,15 leu2 ura3 trp1 ade8 rho+ mit+* [p*RS425 Sod2-3XFLAG-TEV-3XHA: LEU2*] [p*RS416 GAL1 CYB2-TEV Protease: URA3*] | This study |

Table S2: Mass spectrometry data of identified proteins, number of distinct peptides identified for each protein and protein score of each protein from the *erv1-12*-HISPC pulldowns.

| **Protein** | **Accession No.** | **Number of distinct peptides identified** | **Protein score** |
| --- | --- | --- | --- |
| Erv1 | P27882 | \| ENAPQVESR \| \| --- \| \| FDCNFWEK \| \| VDPPDVEQLGR \| \| KVDPPDVEQLGR \| \| IIYDEDGKPCR \| \| MTDNPPQEGLSGR \| \| MTDNPPQEGLSGR \| \| LAGTGALTGEASELMPGSR \| | 362 |
| Aim32 | Q04689 | \| IIDEMYR \| \| --- \| \| LDSLWFGK \| \| FQGETFLR \| \| TDPLDPHIK \| \| HVLLLSPGDR \| \| LSFQDYLSGK \| \| HISVPDLHTR \| \| LLCENLENGK \| | 270 |

**
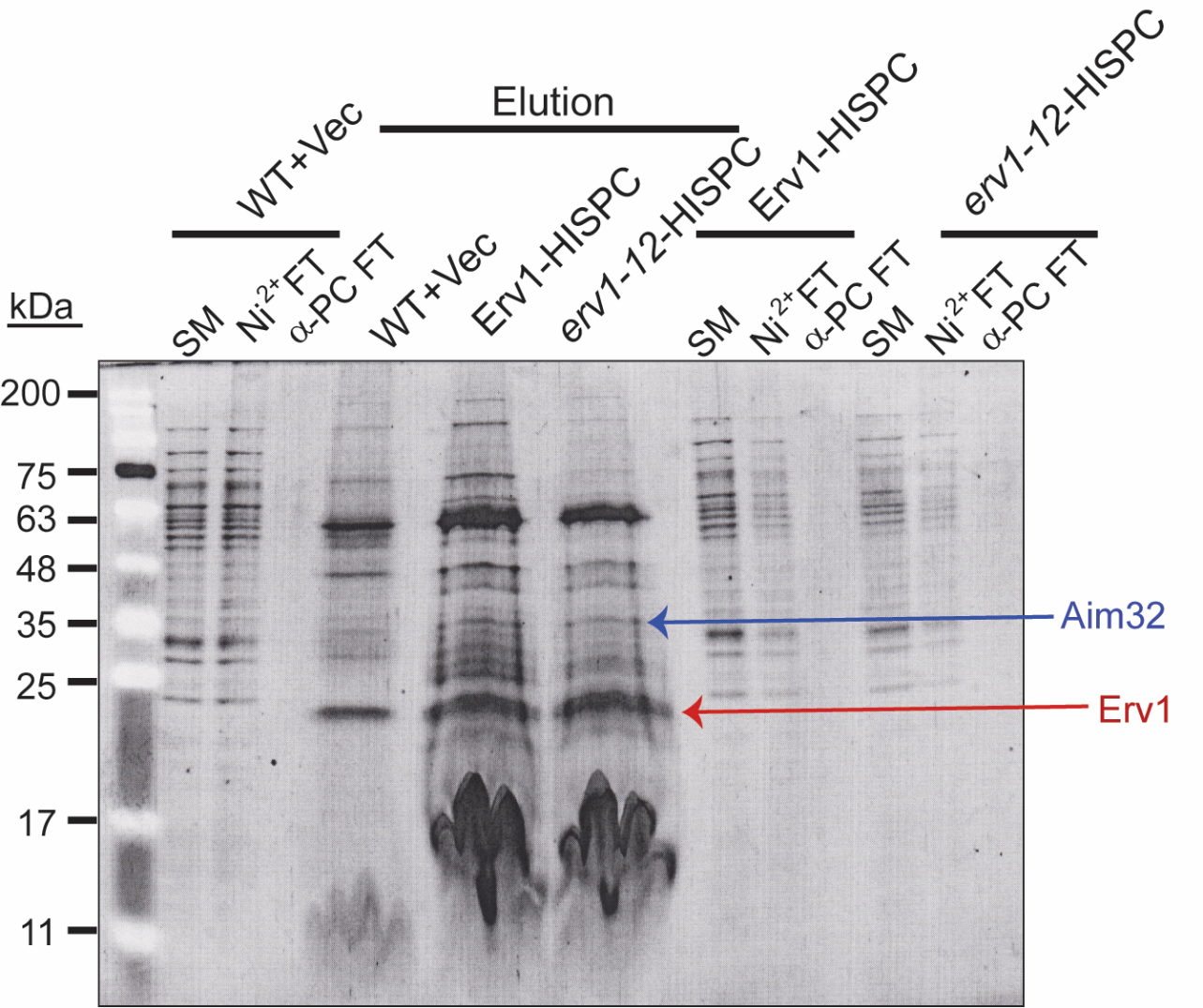
**

**Supplementary Figure S1.** *Aim32 was identified by mass spectrometry as an Erv1 partner protein.* A consecutive-affinity tag [termed CNAP for purification over Ni^2+^-resin followed by anti-Protein C affinity resin] was placed on the C-terminus of Erv1, designated Erv1-HISPC and *erv1-12*-HISPC. The lysate was incubated with Ni^2+^-agarose, eluted with 300 mM imidazole and then incubated with the anti-Protein C resin. After washing, the bound material was eluted, resolved by a 10-15% SDS-PAGE, and stained with SYPRO Ruby solution. The indicated bands (highlighted by red and blue arrows) from WT, Erv1-HISPC, and *erv1-12*-HISPC eluates were excised, digested with trypsin, and identified by LC-MS/MS. (*n* = 3)

**
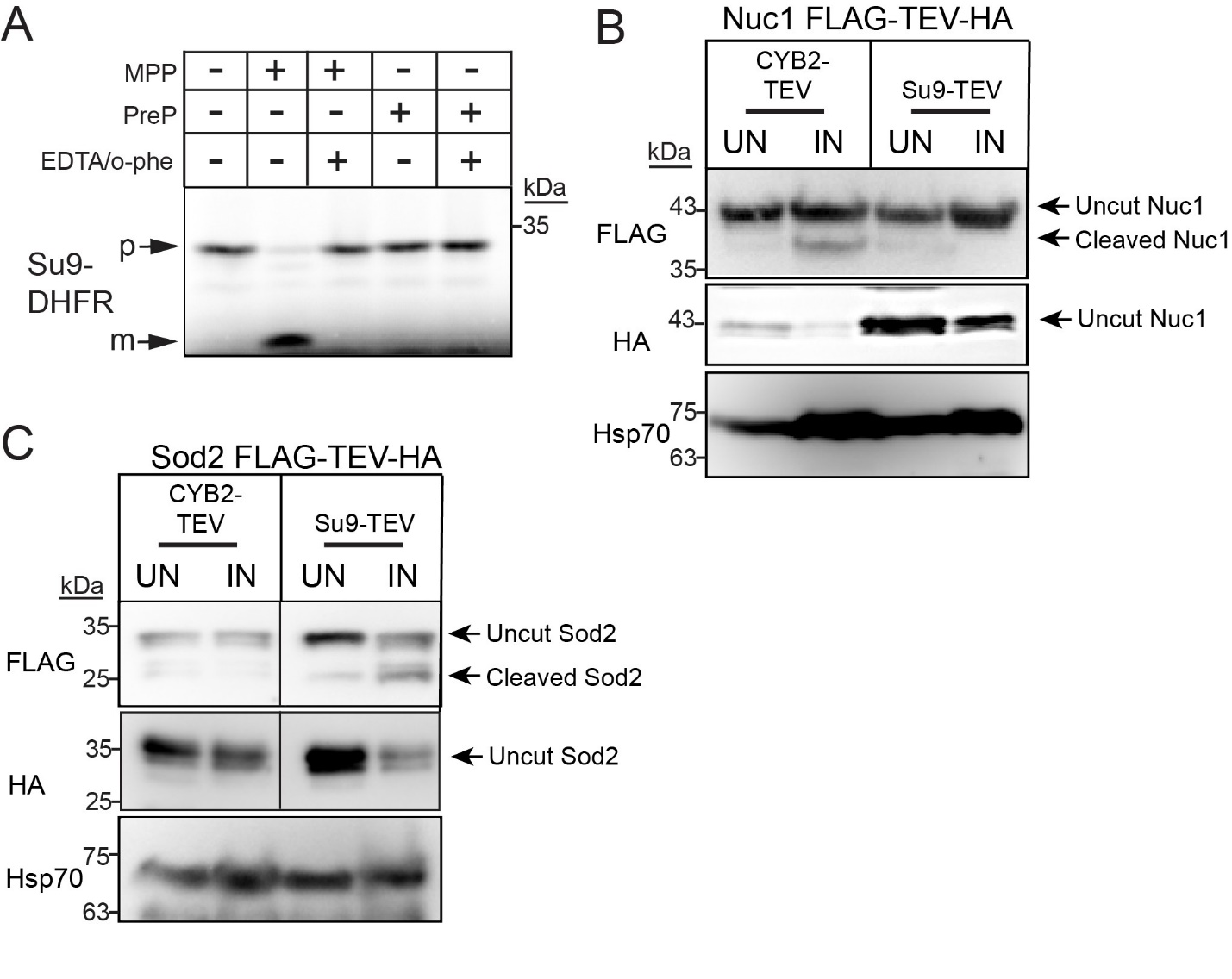
**

**Supplementary Figure S2.** *Control experiments for protease and localization studies.* (A) As in Fig. 2D, the MPP cleavage assay was performed with the addition of 10 μg MPP to radiolabeled Su9-DHFR and the samples were resolved on a 12% Tris-Tricine gel. As a negative control, 10 μg of Presequence protease (Cym1) was included. Chelators EDTA and *o-*phenanthroline were included as inhibitors of MPP and Cym1. p, precursor; m, mature. (B) As in Fig. 2E, WT cells in which IMS-localized Nuc1 was tagged with FLAG-TEV-HA were transformed with plasmids expressing matrix localized Su9-TEV protease or IMS-localized CYB2 [1-220]-TEV protease. Cells were grown in SC-Ura supplemented with 2% galactose (IN; induced), or 2% sucrose (UN; uninduced) and harvested in mid-log phase. Whole cell extracts were analyzed by immunoblotting with antibodies against Hsp70 (loading control) and FLAG and HA for Nuc1. Arrows indicate uncut and TEV cut Nuc1 FLAG-TEV-HA proteins. (C) As in Fig. 2E, WT cells expressing matrix-localized protein Sod2 FLAG-TEV-HA was transformed with plasmids expressing matrix localized Su9-TEV protease or IMS-localized CYB2 [1-220]-TEV protease. Cells were grown in SC-Leu-Ura supplemented with 2% galactose (IN; induced), or 2% sucrose (UN; uninduced) in identical conditions and harvested in mid-log phase. Whole cell extracts were analyzed by immunoblotting with antibodies against Hsp70 (loading control), and FLAG or HA (Sod2 FLAG-TEV-HA). Arrows indicate uncut and TEV protease cut protein.

**
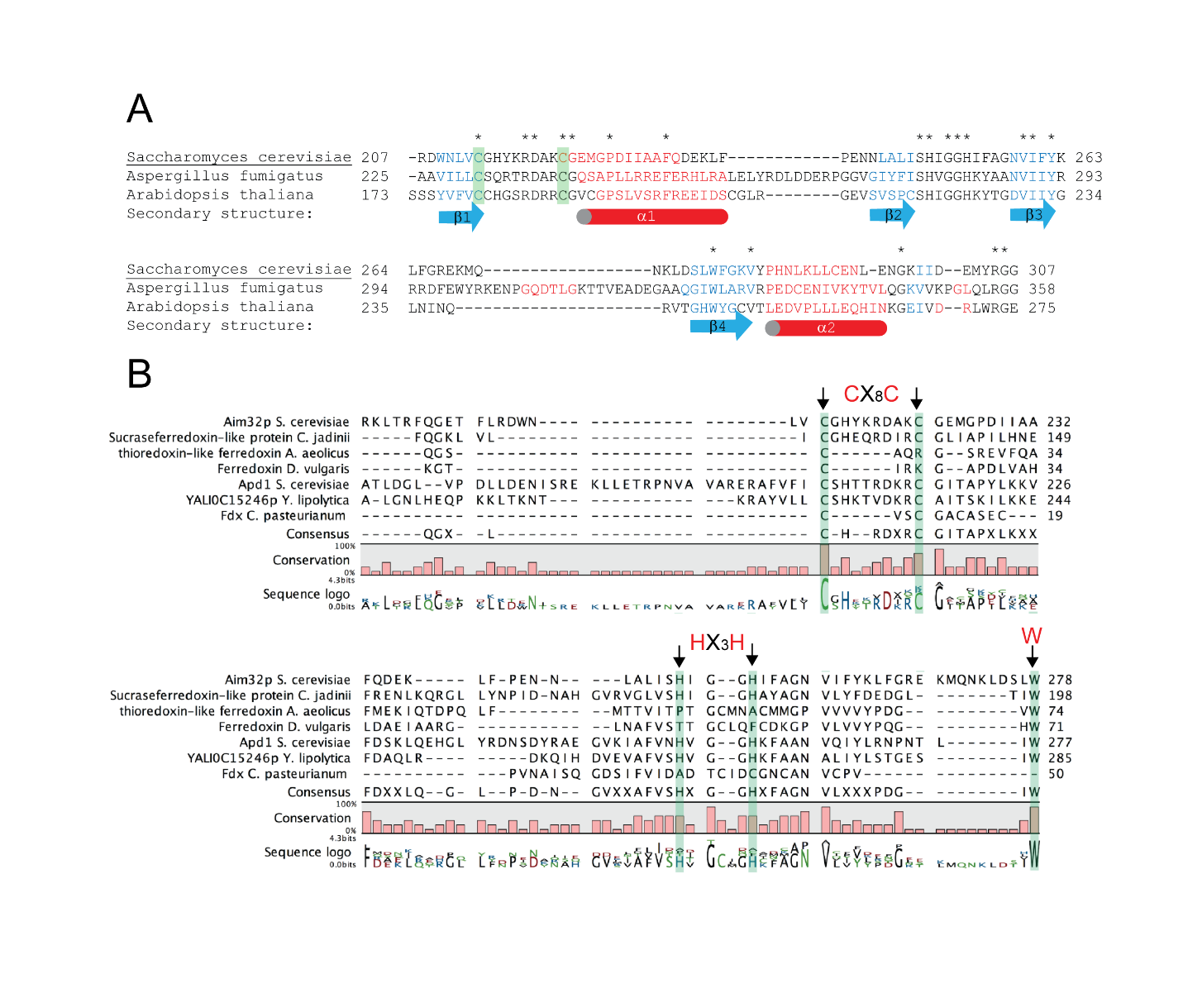
**

**Supplementary Figure S3.** *Aim32 is a thioredoxin-like ferredoxin protein bearing a CX_8_CHX_3_H motif.* (A) Multiple amino acid sequence alignments of three fungal Aim32 homologs depicting homology of the predicted thioredoxin-like fold from amino acids 209-307 (*S. cerevisae*). Sequence alignments were generated using the CLC Workbench software (Qiagen). The helices and strands are labeled in a sequential manner. Starred residues are conserved. (B) Multiple amino acid sequence alignment of Aim32 with other thioredoxin-like ferredoxin (TLF) proteins was performed as in ‘A’. Sequence logos, consensus, and conservation analysis is provided.

**
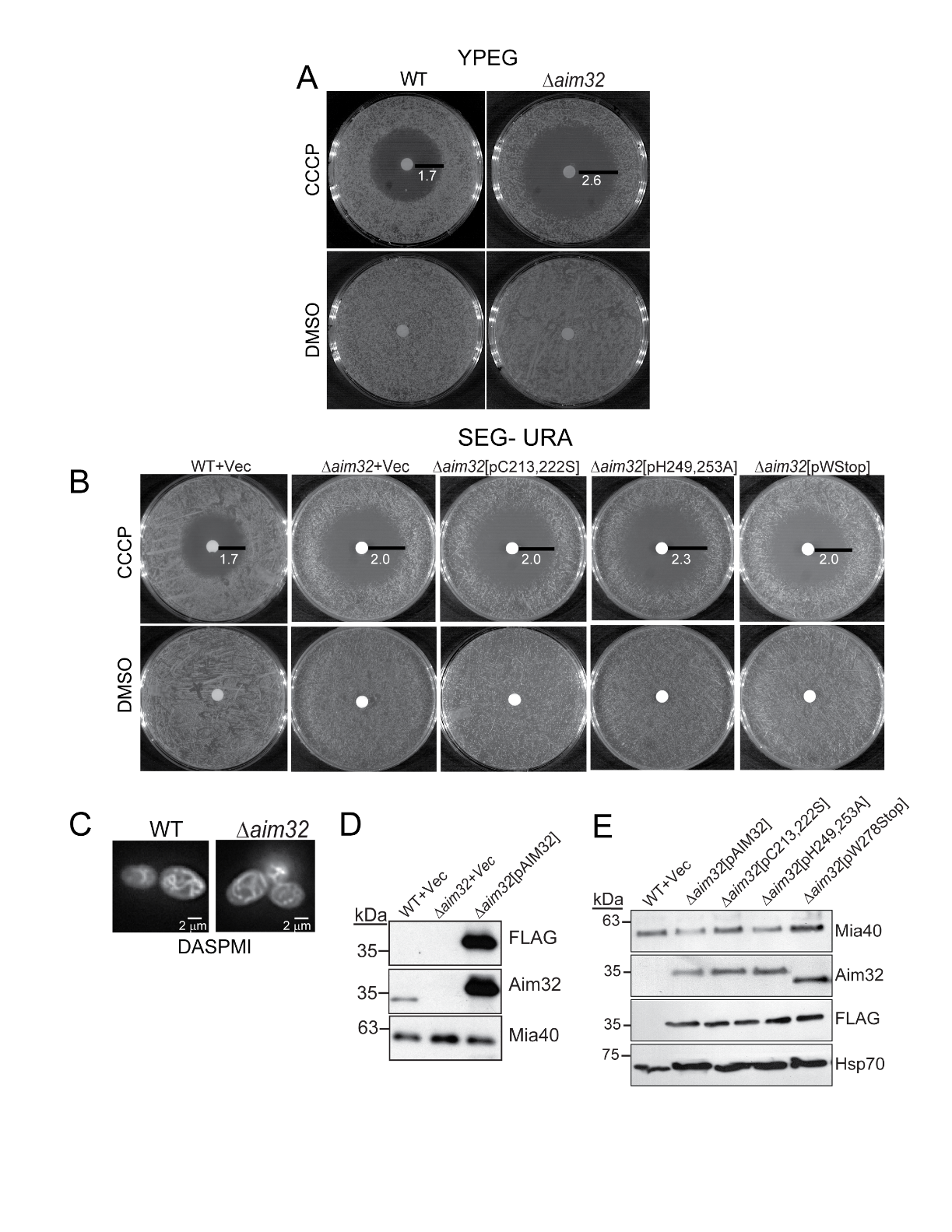
**

**Supplementary Figure S4.** *The CX_8_CHX_3_H motif of Aim32 is crucial to its function.* (A) WT and *Δaim32* cells were plated on YPEG plates and a disk saturated with 0.1 % CCCP or 0.1 % DMSO was placed in the center. Cells were incubated at 30 ^o^C for 2 days and then photographed. The line (measured in cm) indicated the ring in which cells failed to grow. n=3 (B) As in ‘A’, except that growth of *Δaim32* cells and CX_8_CHX_3_H motif variants of Aim32 were analyzed on selective non-fermentable media (SEG-URA). Growth was observed after 2 days of incubation at 30ºC, n=3 (C) WT and *Δaim32* cells were grown at 30^o^C in YPEG media and mitochondria were stained with a potentiometric probe, 2-(4-(dimethylamino)styryl)-1-methylpyridinium iodide (DASPMI). Cells were viewed by fluorescence microscopy. Representative cells are shown. Scale bar, 2μm. (D, E) Expression of Aim32-FLAG fusion proteins was analyzed with antibodies against the FLAG tag and Aim32. Controls are also included.


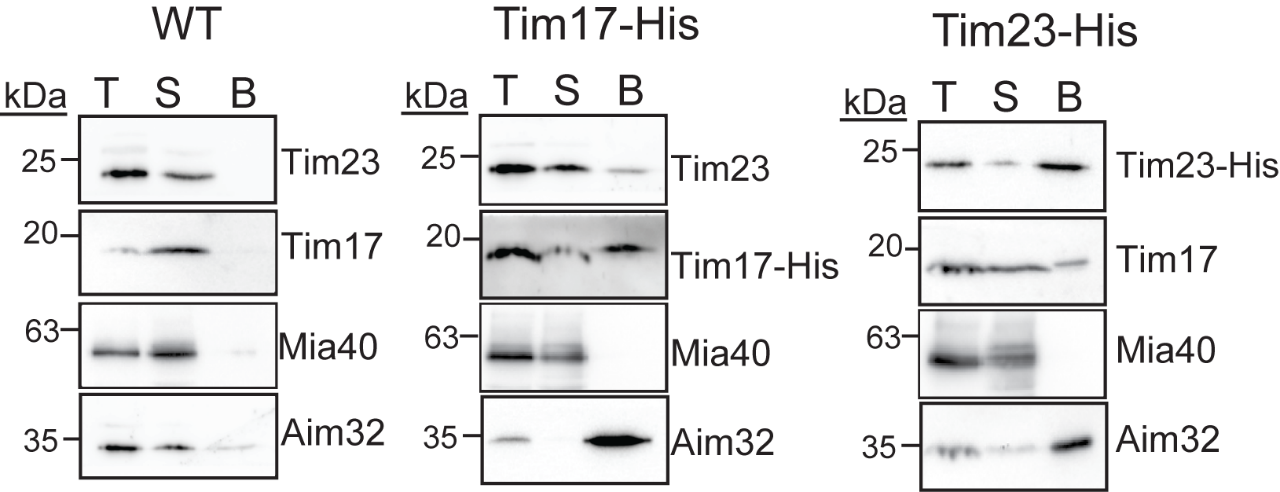


**Supplementary Figure S5.** *Aim32 binds to both Tim17 and Tim23.* Mitochondria from WT (left panel) and strains expressing Tim17 (Tim17-His) (middle panel) or Tim23 (Tim23-His) (right panel) were solubilized in 1% digitonin. As a control, (25 μg) of extract was withdrawn (T), and 500 μg lysate was incubated with Ni^2+^-agarose beads. The beads were washed, and bound protein (B) were eluted. To assess the effectiveness of binding, 25 μg of the unbound protein fraction (S) was also included. Samples were resolved by SDS-PAGE and analyzed by immunoblotting with specific antibodies.

**Supporting References:**

1. Hennig, B., Koehler, H., and Neupert, W. (1983) Receptor sites involved in posttranslational transport of apocytochrome c into mitochondria: specificity, affinity, and number of sites. *Proc Natl Acad Sci U S A* **80**, 4963-4967

2. Dabir, D. V., Leverich, E. P., Kim, S. K., Tsai, F. D., Hirasawa, M., Knaff, D. B., and Koehler, C. M. (2007) A role for cytochrome c and cytochrome c peroxidase in electron shuttling from Erv1. *Embo J* **26**, 4801-4811

3. Hwang, D. K., Claypool, S. M., Leuenberger, D., Tienson, H. L., and Koehler, C. M. (2007) Tim54p connects inner membrane assembly and proteolytic pathways in the mitochondrion. *J Cell Biol* **178**, 1161-1175

4. Dekker, P. J., Martin, F., Maarse, A. C., Bomer, U., Muller, H., Guiard, B., Meijer, M., Rassow, J., and Pfanner, N. (1997) The Tim core complex defines the number of mitochondrial translocation contact sites and can hold arrested preproteins in the absence of matrix Hsp70-Tim44. *EMBO J.* **16**, 5408-5419

5. Neal, S. E., Dabir, D. V., Wijaya, J., Boon, C., and Koehler, C. M. (2017) Osm1 facilitates the transfer of electrons from Erv1 to fumarate in the redox-regulated import pathway in the mitochondrial intermembrane space. *Mol Biol Cell* **28**, 2773-2785
